# TMEM120B strengthens breast cancer cell stemness and accelerates chemotherapy resistance via β1-integrin/FAK-TAZ-mTOR signaling axis by binding to MYH9

**DOI:** 10.1186/s13058-024-01802-z

**Published:** 2024-03-19

**Authors:** Ran Hu, Yu Cao, Yuanyuan Wang, Tingting Zhao, Kaibo Yang, Mingwei Fan, Mengyao Guan, Yuekang Hou, Jiao Ying, Xiaowen Ma, Ning Deng, Xun Sun, Yong Zhang, Xiupeng Zhang

**Affiliations:** 1 Department of Pathology, College of Basic Medical Sciences, First Affiliated Hospital of China Medical University, No.77 Puhe Road, Shenyang North New Area, Shenyang, Liaoning Province 110122 China; 2 Department of Pathology, Shengjing Hospital of China Medical University, Shenyang, China; 3 Department of Surgical Oncology and Breast Surgery, First Affiliated Hospital of China Medical University, Shenyang, China; 4 Department of Anesthesiology, The Fourth Affiliated Hospital, China Medical University, Shenyang, China; 5 Department of Ophthalmology, The First Hospital of China Medical University, Shenyang, China; 6 Department of Immunology, College of Basic Medical Sciences of China Medical University, Shenyang, China; 7 Second Department of Clinical Medicine, China Medical University, Shenyang, China; 8 Department of Breast Surgery, Cancer Hospital of China Medical University, Liaoning Cancer Hospital and Institute, Shenyang, China; 9 Department of Pathology, Cancer Hospital of China Medical University, Liaoning Cancer Hospital and Institute, Shenyang, China

**Keywords:** TMEM120B, MYH9, Focal adhension kinase, Stemness, Breast cancer

## Abstract

**Background:**

Breast cancer stem cell (CSC) expansion results in tumor progression and chemoresistance; however, the modulation of CSC pluripotency remains unexplored. Transmembrane protein 120B (TMEM120B) is a newly discovered protein expressed in human tissues, especially in malignant tissues; however, its role in CSC expansion has not been studied. This study aimed to determine the role of TMEM120B in transcriptional coactivator with PDZ-binding motif (TAZ)-mediated CSC expansion and chemotherapy resistance.

**Methods:**

Both bioinformatics analysis and immunohistochemistry assays were performed to examine expression patterns of TMEM120B in lung, breast, gastric, colon, and ovarian cancers. Clinicopathological factors and overall survival were also evaluated. Next, colony formation assay, MTT assay, EdU assay, transwell assay, wound healing assay, flow cytometric analysis, sphere formation assay, western blotting analysis, mouse xenograft model analysis, RNA-sequencing assay, immunofluorescence assay, and reverse transcriptase-polymerase chain reaction were performed to investigate the effect of TMEM120B interaction on proliferation, invasion, stemness, chemotherapy sensitivity, and integrin/FAK/TAZ/mTOR activation. Further, liquid chromatography–tandem mass spectrometry analysis, GST pull-down assay, and immunoprecipitation assays were performed to evaluate the interactions between TMEM120B, myosin heavy chain 9 (MYH9), and CUL9.

**Results:**

TMEM120B expression was elevated in lung, breast, gastric, colon, and ovarian cancers. TMEM120B expression positively correlated with advanced TNM stage, lymph node metastasis, and poor prognosis. Overexpression of TMEM120B promoted breast cancer cell proliferation, invasion, and stemness by activating TAZ-mTOR signaling. TMEM120B directly bound to the coil-coil domain of MYH9, which accelerated the assembly of focal adhesions (FAs) and facilitated the translocation of TAZ. Furthermore, TMEM120B stabilized MYH9 by preventing its degradation by CUL9 in a ubiquitin-dependent manner. Overexpression of TMEM120B enhanced resistance to docetaxel and doxorubicin. Conversely, overexpression of TMEM120B-∆CCD delayed the formation of FAs, suppressed TAZ-mTOR signaling, and abrogated chemotherapy resistance. TMEM120B expression was elevated in breast cancer patients with poor treatment outcomes (Miller/Payne grades 1–2) than in those with better outcomes (Miller/Payne grades 3–5).

**Conclusions:**

Our study reveals that TMEM120B bound to and stabilized MYH9 by preventing its degradation. This interaction activated the β1-integrin/FAK-TAZ-mTOR signaling axis, maintaining stemness and accelerating chemotherapy resistance.

**Supplementary Information:**

The online version contains supplementary material available at 10.1186/s13058-024-01802-z.

## Background

Breast cancer is the most commonly diagnosed malignancy worldwide [1]. Hormonal therapies and targeted treatment have improved the overall survival of patients with certain breast cancer subtypes. However, surgery after receiving neoadjuvant chemotherapy is a better treatment strategy for breast cancer, as it can shrink tumor size and downgrade the tumor, node, and metastasis (TNM) stage [2–4]. Anthracyclines such as doxorubicin and taxanes such as docetaxel and platinum-based drugs are widely used therapeutic options for neoadjuvant chemotherapy; however, subsequent chemotherapy resistance limits effective cancer treatment.

Cancer stem cell (CSC) expansion is considered the primary cause of chemotherapy resistance [5]. Increasing evidence indicates that numerous signaling pathways, such as the JAK/STAT, Hedgehog, Wnt, Notch, PI3K/AKT, and Hippo pathways, are involved in modulating CSC maintenance, [6–12].

Hippo signaling pathway is well investigated on determining cell proliferation and stemness [13, 14], whose activation results in sequential phosphorylation to MST(Mammalian STE20-like protein kinase), LATS (large tumour suppressor kinase), YAP (Yes-associated protein) and TAZ (Transcriptional coactivator with PDZ-binding motif) [15–17]. Phosphorylated YAP/TAZ are distributed to ubiquitylation and proteasomal degradation, inversely, YAP/TAZ translocate into nucleus and accelerated transcriptional program as co-activators [18–23]. YAP/TAZ also serve as core nuclear effectors of mechanical force signaling transduction upon ECM (Extracellular matrix) remolding [24, 25]. Focal adhesion kinase (FAK) is reported to be crucial in modulating integrin-dependent cell motility, which induces mechanical force transduction, thus raise nuclear YAP/TAZ [26, 27]. Both YAP/TAZ and FAK signaling were proven to play irreplaceable roles in maintaining cancer cell stemness and accelerate chemotherapy resisitance [28–32].

Myosin heavy chain 9 (MYH9) is a widely expressed cytoplasmic myosin that binds to actin, converts chemical energy into mechanical force by hydrolyzing ATP, and promotes tumor development by participating in cell proliferation, migration, stem cell differentiation, and signal transduction [33, 34]. Zhong et al. reported that MYH9 can be activated to accelerate the adhesion plaque assembly and transmit mechanistic force signals from the cell edge to the nucleus by modulating FAK and integrin [35].

Transmembrane 120B (TMEM120B) localizes on chromosome 12q24.31 and is composed of six transmembrane domains and a coil-coil domain [36]. Its homolog TMEM120A, also known as NET29, is crucial during adipogenesis and is expressed in both white and brown adipose tissues [37]. The conditional knockout of TMEM120A in mouse adipocytes leads to lipodystrophy [38]. Deletion of TMEM120A was also reported to enhance chemotherapy sensitivity of colon cancer cells [39]. However, to date, the expression patterns and biological functions of TMEM120B in human tissues, especially in malignant tumors, have not been reported. Our bulk RNA-sequencing and MS (Mass spectrum) data revealed that TMEM120B may bind to MYH9 and activate hippo and FAK signaling thus mediated CSC expansion and chemotherapeutic resistance.

## Methods

### Patients and clinical specimens

The study protocol was approved by the Institutional Review Board of the China Medical University. All participants provided written informed consent, and the study was conducted in accordance with the principles of the Declaration of Helsinki. Detailed descriptions of patient information have been described previously [40] and can be found in Additional file 1.

### Functional enrichment analyses

This was performed as described by Li et al. [41]. Detailed descriptions are provided in Additional file 1.

### Cell culture

The human breast cancer cell lines MCF-10 A, MCF-7, MDA-MB-231, MDA-MB-453, BT-474, and SK-BR-3 were obtained from the Shanghai Cell Bank (Shanghai, China), and ZR75-1 and MDA-MB-468 were purchased from Procell Life Science and Technology (Wuhan, China). All cell lines were authenticated using short tandem repeat DNA profiling with no more than 10 passages and no mycoplasma contamination. Cells were cultured in RPMI 1640 medium (Invitrogen, Carlsbad, CA, USA) containing 10% fetal calf serum (Invitrogen), 100 IU/mL penicillin, and 100 µg/mL streptomycin (Sigma-Aldrich, St. Louis, MO, USA). The cells were cultured in sterile culture dishes at 37 °C in a 5% CO_2_ atmosphere.

### Reagents

pCMV6-TMEM120B-Myc, pCMV6-TMEM120B-ΔCCD-Myc, pCMV6-TMEM120B-ΔTMD1-Myc, pCMV6-TMEM120B-ΔTMD2-Myc, Plv3-U6-TMEM120B-sgRNA-cas9-GFP #1 (ACGCCAGTACGAACACCTGGGGG), #2 (GGACGTCTTCTTCGACATGGAGG), and control sgRNA (GCACTACCAGAGCTAAC-TTCA), pCMV6-mCherry-MYH9-FLAG, pCMV6-mCherry-MYH9-SH3-FLAG, pCMV6-mCherry-MYH9-delCCD-FLAG, pCMV6-mCherry-MYH9-IQCCD-FLAG, pCMV6-CUL9-HA, FAK siRNA, MYH9 siRNA were purchased from MiaoLingBio (Wuhan, China). TAZ siRNA (sc-38,568) and negative control (NC) siRNA (sc-37,007) were obtained from Santa Cruz Biotechnology. Lipo3000 (Invitrogen, Carlsbad, CA, USA) was used for transfection. Rapamycin (AY-22,989), PF562271(VS-6062), MG132 (HY-13,259), cycloheximide (20ug/ml, HY-12,320), doxorubicin (HY-15,142 A), and docetaxel (HY-12,053 A) were purchased from MedChemExpress (Monmouth Junction, NJ, USA).

### Western blotting and immunoprecipitation

Western blotting and immunoprecipitation were performed as described by Zhang et al. [42]. Human Phospho-Kinase Array Kit (Catalog #: ARY003C) were purchased form R&D systems (Minneapolis, MN, USA), which was performed according to the manufacturer’s instructions. Detailed descriptions of the antibodies used are provided in Additional file 1.

### MTT and colony formation assay

The MTT and colony formation assays were performed as described by Zhang et al. [42]. Detailed descriptions are provided in Additional file 1.

### EdU assay

TMEM120B-overexpressing-MCF-7 and SK-BR-3 or TMEM120B-knocking out-MDA-453 and MDA-231 cells were cultured with an EdU solution of 20 µM (Cellorlab, Shanghai, China) for 1 h, after which all steps were performed as described by Zhang et al. [42]. The cells were incubated with 20 µM EdU solution (Cellorlab, Shanghai, China) for 1 h after overexpressing or knocking out TMEM120B.

### Wound healing and transwell assay

Assays were performed as described by Zhang et al. [43]. Detailed descriptions are provided in Additional file 1.

### Three-dimensional invasion

Cells were embedded at a density of 1.5 cells/µL into neutralized, fibrillar rat-tail collagen I (Corning, No.354,236), into 24-well glass-bottomed plates, on a 37 °C heating block. Collagen gels were allowed to polymerize at 37 °C for approximately 1 h, after which media Dulbecco’s modified eagle medium (DMEM)-F12 (Gibco, No.10,565–018), 1% insulin-transferrin-selenium (Gibco, No.51,500–056, Invitrogen, Carlsbad, CA, USA), 1% penicillin-streptomycin (Sigma, No.P4333), and 2.4 nM FGF2 (Sigma, No.F0291) were added to the wells. Tumor cells invaded collagen I over 24–48 h.

### Nocodazole (NZ) model system

The experiment followed the method published by Nader et al. [44]. In brief, SK-BR-3 cells with ectopic TMEM120B or TMEM120B-∆CCD were grown on glass coverslips. After 48 h, the cells were treated with 10 µM NZ for 4 h to completely depolymerize microtubules (MTs). Subsequently, the NZ was removed and washed with serum-free medium, leading to MT repolymerization followed by focal adhesion (FA) disassembly. The cells were fixed for 1 h in 4% paraformaldehyde for 30 min, followed by immunofluorescence staining. Integrin β1 and p-FAK levels on the cell membrane were quantified by their mean fluorescent signal intensity using ImageJ (National Institute of Health, Bethesda, MD, USA).

### Immunofluorescence staining

MCF-7, SK-BR-3, MD-231, MDA-453, MDA-468 cells were seeded in 24-well plates for 24 h, washed with PBS, fixed with 4% paraformaldehyde for 30 min, permeabilized with 0.5% Triton X-100 for 10 min, and stained with the indicated antibodies. Images were captured using laser scanning confocal microscopy (Carl Zeiss, Thornwood, NY, USA).

### Sphere formation assay

For the first round of sphere formation assay, 1 × 10^3^ TMEM120B-overexpressing-MCF-7 and SK-BR-3 or TMEM120B-knocking out-MDA-453 and MDA-231 cells were cultured in a 24-well low-attachment surface polystyrene culture plate (Costar, Cambridge, MA, USA) using serum-free DMEM-F12 (Invitrogen, Carlsbad, CA, USA), containing 1×B27 (Invitrogen, Carlsbad, CA, USA), 20 ng/mL EGF (BD Bioscience, San Jose, CA, USA), and 4 mg/mL insulin (Sigma, St. Louis, MO, USA) at 37 °C and 5% CO_2_ for 10–14 days. Mammospheres with a diameter > 75 μm in five randomly selected fields were counted. For the second round of the sphere formation assay, the spheroids were centrifuged at 800 g and collected, after which pancreatic enzymes were added for full digestion and blowing. The shattered spheroids were filtered using a 70 μm single-cell sieve, leaving only the spheroids that had been dispersed into single cells. After counting, the spheroids were added to a 24-well low-attachment surface polystyrene culture plate (Corning, NY, USA), and the rest were subjected to the above procedure.

### Flow cytometry

The ALDEFLUOR assay was performed according to the manufacturer’s instructions (STEMCELL Technologies, Vancouver, British Columbia, Canada). Cell pellets were re-suspended in 0.5 mL of ALDEFLUOR™ Assay Buffer and stored on ice or at 4 °C. Samples were analyzed using a MoFlo Astrios or CytoFlex (Beckman Coulter, USA) equipped with a 488/513 nm channel for ALDEFLUOR or FITC.

### RNA extraction and real-time PCR

These assays were performed as described by Zhang et al. [43]. Primer sequences are shown in Additional file 2.

### LC-MS/MS analysis

TMEM120B or TMEM120B-ΔCCD overexpression proteins of SK-BR-3 cells in the gel pieces from co-immunoprecipitation (co-IP) were analyzed by nano-LC-MS/MS on a Q Exactive mass spectrometer (Thermo Fisher Scientific) coupled with an Easy nLC system (Invitrogen, Carlsbad, CA, USA). Raw MS/MS data were converted into MGF format using Proteome Discoverer 1.4 (Invitrogen, Carlsbad, CA, USA). Peptide identification was performed using Mascot software (Version 2.3.01, Matrix Science, UK) with the UniProt database search algorithm and an integrated FDR analysis function. The data were used to conduct searches against a protein sequence database downloaded from the 2021_uni_mus (128,510 sequences; 62,817,431 residues). The MS/MS spectra were searched against a decoy database to estimate the false discovery rate (FDR ˂ 0.05) for peptide identification.

### RNA-sequencing

Total RNA was extracted from control and TMEM120B-knockout MDA-MB-453 cells using TRIzol reagent (Takara, Kyoto, Japan) according to the manufacturer’s instructions. RNA purity was determined using a Nano Photometer spectrophotometer (IMPLEN, Westlake Village, USA). cDNA libraries were constructed from 1 µg of total RNA using a PCR-cDNA Sequencing Kit (SQK-PCS109; Nanopore Technologies, Oxford, UK) according to the manufacturer’s protocol. Genes with an FDR < 0.05 and fold change ≥ 2.0, identified using DESeq, were designated as “differentially expressed.” All operations were performed using biomarker technologies (www.bionarker.com.cn).

### GST pull-down assay

This assay was performed as described by Han et al. [45]. TMEM120B protein coupled to a GST label was induced in *Escherichia coli* BL21 (30 °C, 3 h, 200 rpm) and purified using standard procedures. The purified protein was recombined with glutathione sepharose (GE Healthcare, Waukesha, WI, USA) magnetic beads and then incubated overnight with SK-BR-3-TMEM120B-Full length or -TMEM120B-ΔCCD cell lysates transfected with Myc-MYH9 plasmid at 4 °C. Finally, the complexes were detected using western blotting and Coomassie Brilliant Blue staining.

### Transplantation of tumor cells into nude mice and limiting dilution analysis

The animals were treated according to the National Institutes of Health Guidelines for the Care and Use of Laboratory Animals (NIH Publication No. 8023, revised 1978). Nude mice were treated according to the experimental animal ethics guidelines issued by the China Medical University (CMU2021731). The detailed descriptions are provided in Additional file 1. For the limiting dilution injection, 8-week-old nude female recipient mice (for MDA-MB-231-TMEM120B-KO or SK-BR-3-overexpressing TMEM120B or TMEM120B-ΔCCD cells) were anesthetized with isoflurane. MDA-MB-231 or SK-BR-3 breast cancer cells were suspended at a different density into a 1:1 mixture of DMEM and Matrigel (BD Biosciences). Cells were injected into nude mice at 3$\times$10^4^, 1$\times$10^4^, 3$\times$10^3^, or 1$\times$10^3^ cells.This data was collected and analyzed by limiting dilution analysis using ELDA software (http://bioinf.wehi.edu.au/software/elda/) [46].

### Statistical analyses

All data were analyzed using SPSS version 22.0 (Chicago, IL, USA). The chi-square test was used to evaluate the correlation between TMEM120B expression and clinicopathological factors. Kaplan–Meier survival curves were plotted, and the log-rank test was performed. Spearman’s correlation analysis was performed to examine the correlation between TMEM120B, p-mTOR, TAZ, and SOX2 expression respectively. All clinicopathological parameters were included in the Cox regression model and assessed by univariate analysis using the enter method. The student’s t-test was used to analyze differences between the groups. One-way analysis of variance (ANOVA) was used to compare multiple groups. All experiments were performed in triplicate. A P-value less than 0.05 was considered statistically significant.

## Results

### Elevated TMEM120B expression in breast cancer correlated with advanced TNM stage, positive lymph node metastasis, and poor prognosis

**Fig. 1:**
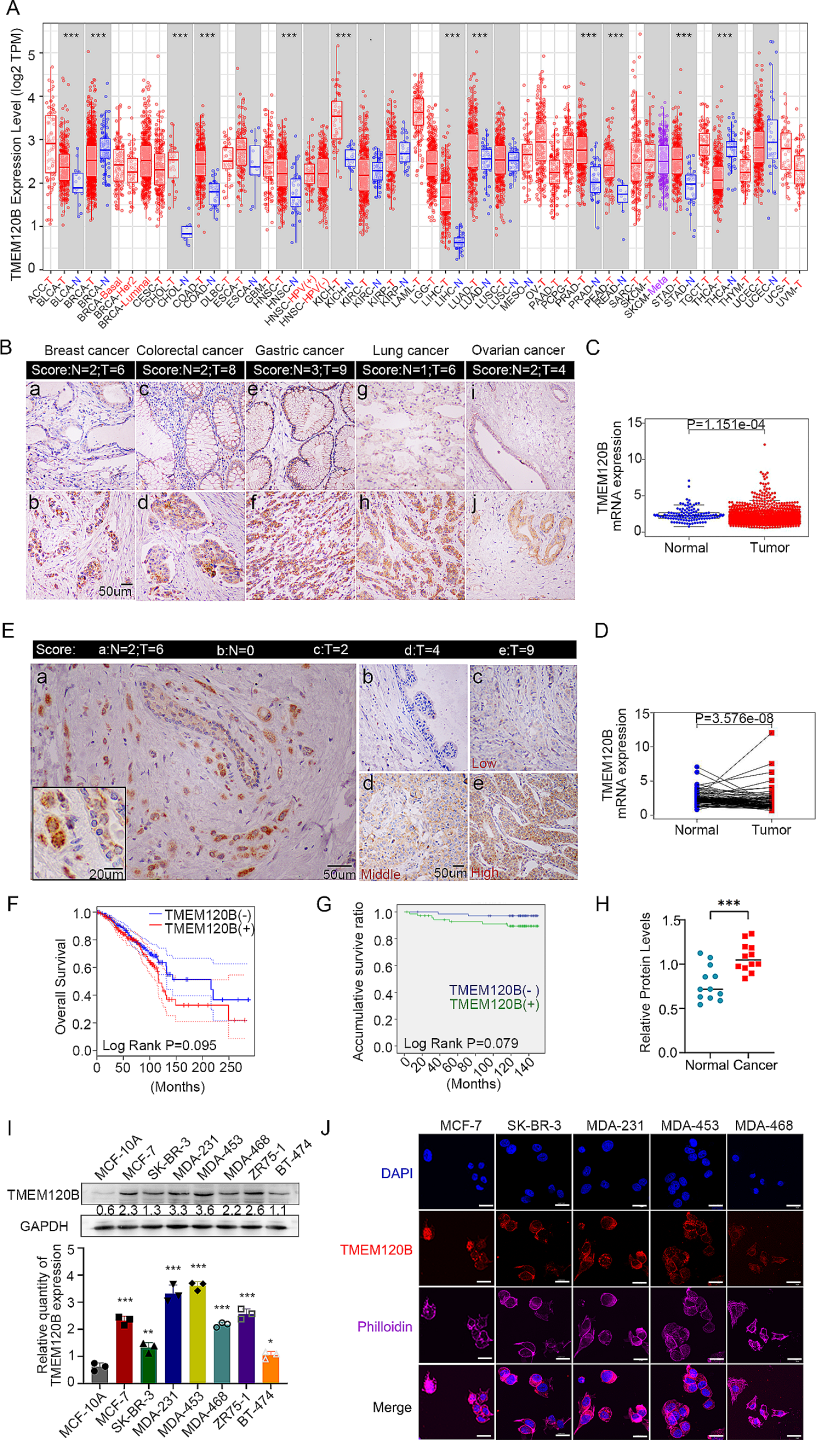
TMEM120B was highly expressed in breast cancer specimens and cell lines. (**A**) TCGA database was assessed to explore the mRNA expression of TMEM120B in pan-cancer and normal tissues, N for Normal, T for Tumor, Meta for metastasis. (**B**) Representative images of immunohistochemistry staining of TMEM120B in normal breast epithelial cells (a), normal intestinal epithelial cells (c), normal gastric epithelial cells (e), normal lung epithelial cells(g), normal ovarian epithelial cells (i) breast cancer epithelial cells (b), colon cancer epithelial cells (d), gastric carcinoma epithelial cells (f), lung cancer epithelial cells (h) and ovarian cancer epithelial cells (j), N for Normal, T for Tumor. (**C**-**D**) *TMEM120B* mRNA levels were identified between non-cancerous and cancerous tissues using the TCGA database (**E**) Representative images of immunohistochemistry staining of TMEM120B in (a) both normal and cancerous tissues in the same specimen, (b) adjacent normal tissue and breast cancer with diverse staining (c, weak, d, moderate, e, strong), N for Normal, T for Tumor. (**F**) Kaplan–Meier curves showed a correlation between mRNA expression of *TMEM120B* and overall survival in breast cancer patients. (**G**) Kaplan–Meier curves showing a correlation between TMEM120B protein expression and overall survival of patients with breast cancer. (**H**) TMEM120B protein level in 16 pairs of freshly isolated samples from patients with breast cancer was analyzed by western blotting (**I**) The protein expression of TMEM120B in breast cancer cell lines and normal breast cells. (**J**) Immunofluorescence assay was used to evaluate the subcellular localization of TMEM120B in breast cancer cells (scale bar = 20 μm). Quantification data are expressed as mean ± SD of three independent experiments (t-test, two-sided, *P < 0.05, **P < 0.01, ***P < 0.001)

First, we used The Cancer Genome Atlas (TCGA) database to explore the mRNA expression of TMEM120B in pan-cancer and normal tissues and found that TMEM120B was highly expressed in most cancerous tissues than in non-cancerous ones; however, breast cancer was excluded (Fig. 1A). Subsequent immunohistochemical (IHC) staining of 20 cases of lung cancer, 29 cases of breast cancer, 21 cases of gastric carcinoma, 24 cases of colon cancer, 20 cases of ovarian cancer, and paired normal tissues suggested that TMEM120B expression was elevated in all malignant cancerous tissues compared with that in normal tissues (Fig. 1B:a-j and Table 1). Moreover, we examined *TMEM120B* mRNA expression specifically in breast cancer based on the gene expression profiling interactive analysis (GEPIA) database and found that *TMEM120B* mRNA expression was significantly higher in breast cancer than in normal tissues, both in paired and unpaired breast cancer specimens (Fig. 1C and D). Next, we performed IHC staining of 140 breast cancer and 42 normal samples and found that the positive rate of TMEM120B in breast cancer tissues(50.7%,71/140) was significantly higher than that in the paired non-cancerous tissues (23.8%,10/42, *P* < 0.001,Fig. 1E: a-e), with a cytosolic positive expression rate of 47.1%(66/140) (Fig. 1E: a-e), and a nuclear expression rate of 3.5% (5/140; Additional file 3: Fig. S1A). Subsequent statistical analysis indicated that total and cytosolic TMEM120B expression positively correlated with advanced TNM stage (*P* = 0.011,*P* = 0.015) and lymph node metastasis (*P* = 0.006,*P* = 0.007) but not with age or triple-negative breast cancer, however, nuclear TMEM120B revealed no visible correlation with clinicopathologic factors(*P* > 0.05, Table 2). Bioinformatics analysis also suggested that TMEM120B expression was higher in patients with advanced SBR grade and positive distant metastasis than lower grade and negative groups (Additional file 3: Fig. S1B-C). Whereas it reveal no obvious differences for *TMEM120B* RNA among diverse subtypes of breast cancer(Additional file 3: Fig.S1D).Kaplan–Meier analysis revealed that both TMEM120B mRNA and protein levels were higher in patients with a poor prognosis than better ones (*P* = 0.095 and *P* = 0.079, Fig. 1F-G). However, Cox univariate analysis revealed that TMEM120B expression was not an independent prognostic factor in patients with breast cancer (Table 3). Additionally, western blotting was performed on fresh breast cancer and paired normal tissue samples. The results suggested that TMEM120B protein levels were higher in cancerous samples than in non-cancerous samples (Fig. 1H and Additional file 3: Fig. S1E), which was consistent with the IHC staining results.

**Table 1:** Expression of TMEM120B in various epithelial malignancies

Tissue sources	N	positive	negative	χ^2^	P
Breast carcinoma	29	17	12	7.137	0.009
Normal breast tissue	15	4	11		
Colon carcinoma	24	14	10	6.337	0.013
Normal colon tissue	13	2	11		
Lung carcinoma	20	11	9	4.915	0.029
Normal Lung tissue	16	3	13		
Gastric carcinoma	21	13	8	4.859	0.031
Normal stomach tissue	13	3	10		
Ovarian carcinoma	20	12	8	4.970	0.029
Normal ovary tissue	14	3	11		

**Table 2:** Correlation of the expression of TMEM120B with clinicopathological features in 140 cases of breast cancer

Clinicopathological	Total TMEM120B					Cytosolic expression				Nuclear expression			
factors	N	(+)	(-)	χ2	*P*	N	(+)	(-)	*P*	N	(+)	(-)	*P*
Age (years)													
<51	74	37	37	0.032	0.867	70	34	36	0.727	4	3	1	0.524
≥51	66	34	32			61	32	29		5	2	3	
TNBC													
Yes	63	33	30	0.127	0.737	58	31	27	0.599	5	2	3	0.524
No	77	38	39			73	35	38		4	3	1	
TNM classification													
I + II	95	41	54	6.751	0.011	89	38	51	0.015	6	3	3	1.000
III	45	30	15			42	28	14		3	2	1	
Lymph node metastasis													
Positive	55	36	19	7.875	0.006	50	33	17	0.007	5	3	2	1.000
Negative	85	35	50			81	33	48		4	2	2	
Subcellular localization													
Cytoplasm	131	66	65	0.090	1.000								
Nucleus	9	5	4										

**Table 3:** Summary of Cox univariate and multivariate regression analysis of the association between clinicpathological features and overall survival in 140 cases of breast cancer

Clinicopathological	Regression	Wald chi-	P	Risk	95% CI	
feature	coefficient	square test		ratio	Lower	Upper
Univariate analysis						
Age	-0.586	0.688	0.407	0.556	0.139	2.224
Triple-negative	0.494	0.488	0.485	1.639	0.410	6.552
TNM classification	2.129	7.048	0.008	4.787	1.746	40.505
Lymph node metastasis	2.652	6.247	0.012	2.883	1.772	113.418
TMEM120B expression	0.923	5.092	0.101	2.516	1.129	5.607

We examined the expression and subcellular localization of TMEM120B in seven breast cancer cell lines and one normal breast cancer cell line (MCF-10 A). Western blot analysis indicated that TMEM120B expression was higher in all detected breast cancer cell lines than in MCF-10 A cells (Fig. 1I). Immunofluorescence staining also indicated that TMEM120B exhibited both cytosolic and nuclear localization in breast cancer cell lines (Fig. 1J).

### Overexpression of TMEM120B accelerated breast cancer proliferation and invasion both in vitro and in vivo

**Fig. 2:**
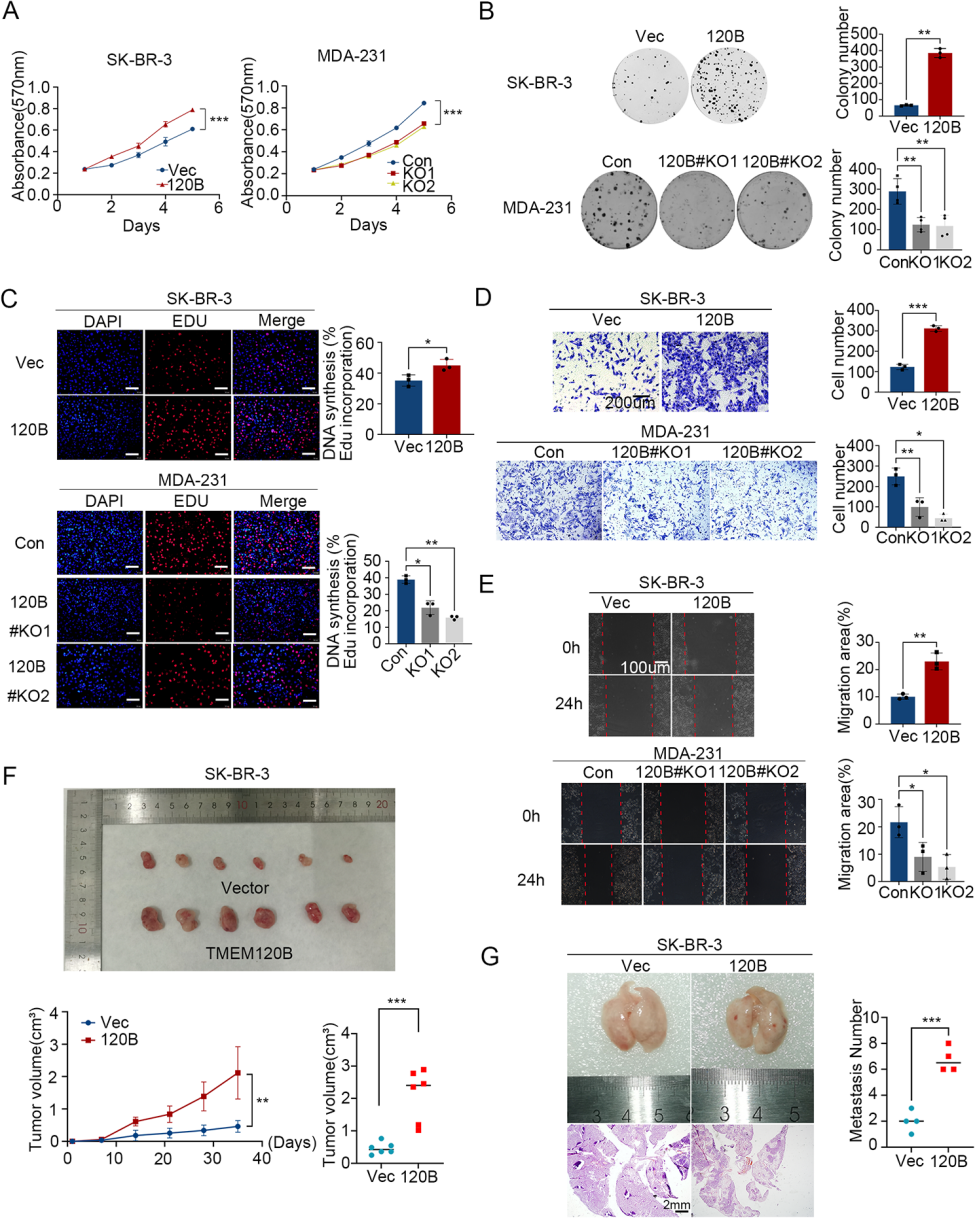
Overexpression of TMEM120B promoted breast cancer cell proliferation and invasion both in vitro and in vivo. The MTT assay (**A**), colony formation assay (**B**), and EdU assay (**C**, scale bar = 100 μm) were performed to examine the effects on the proliferation of after overexpressing or silencing TMEM120B in SK-BR-3 or MDA-231 cells. Transwell (**D**) and wound healing (**E**) assays were used to assess the effects of TMEM120B-myc, TMEM120B sgRNA, and the control on cell invasion and migration in SK-BR-3 and MDA-231 cells. Representative examples of explanted tumors (**F**) and lung metastases (**G**) in the negative SK-BR-3-control (NC) and SK-BR-3- overexpressing TMEM120B groups. Quantification data are expressed as mean ± SD of three independent experiments (t-test, two-sided, *P < 0.05, **P < 0.01, ***P < 0.001)

We first overexpressed TMEM120B in both MCF-7 and SK-BR-3 cells or deleted TMEM120B with two different sgRNAs using CRISPR-Cas9 in MDA-231 and MDA-453 cells (Additional file 3: Fig. S2A). The results of the MTT assay (Fig. 2A and Additional File 3: Fig. S2B), colony formation (Fig. 2B; Additional file 3: Fig. S2C), and EdU (Fig. 2C and Additional file 3: Fig. S2D) assays indicated that the overexpression or deletion of TMEM120B may promote or abrogate MCF-7, SK-BR-3,MDA-231 and MDA-453 cells proliferation, respectively. Transwell assay (Fig. 2D and Additional file 3: Fig. S2E) and wound healing (Fig. 2E and Additional file 3: Fig S2F) assays also revealed that migration and invasion were enhanced or suppressed by the overexpression or inhibition of TMEM120B. Additionally, xenograft assays revealed that tumor volumes significantly increased in SK-BR-3 cells overexpressing TMEM120B (Fig. 2F), whereas the number of lung metastases visibly increased in the ectopic TMEM120B group, however there were no obvious changes in metastasis of liver, brain, kidney and heart (Fig. 2G, Additional file 3: Fig. S2G).

### Overexpression of TMEM120B enhanced stemness of breast cancer cells

**Fig. 3:**
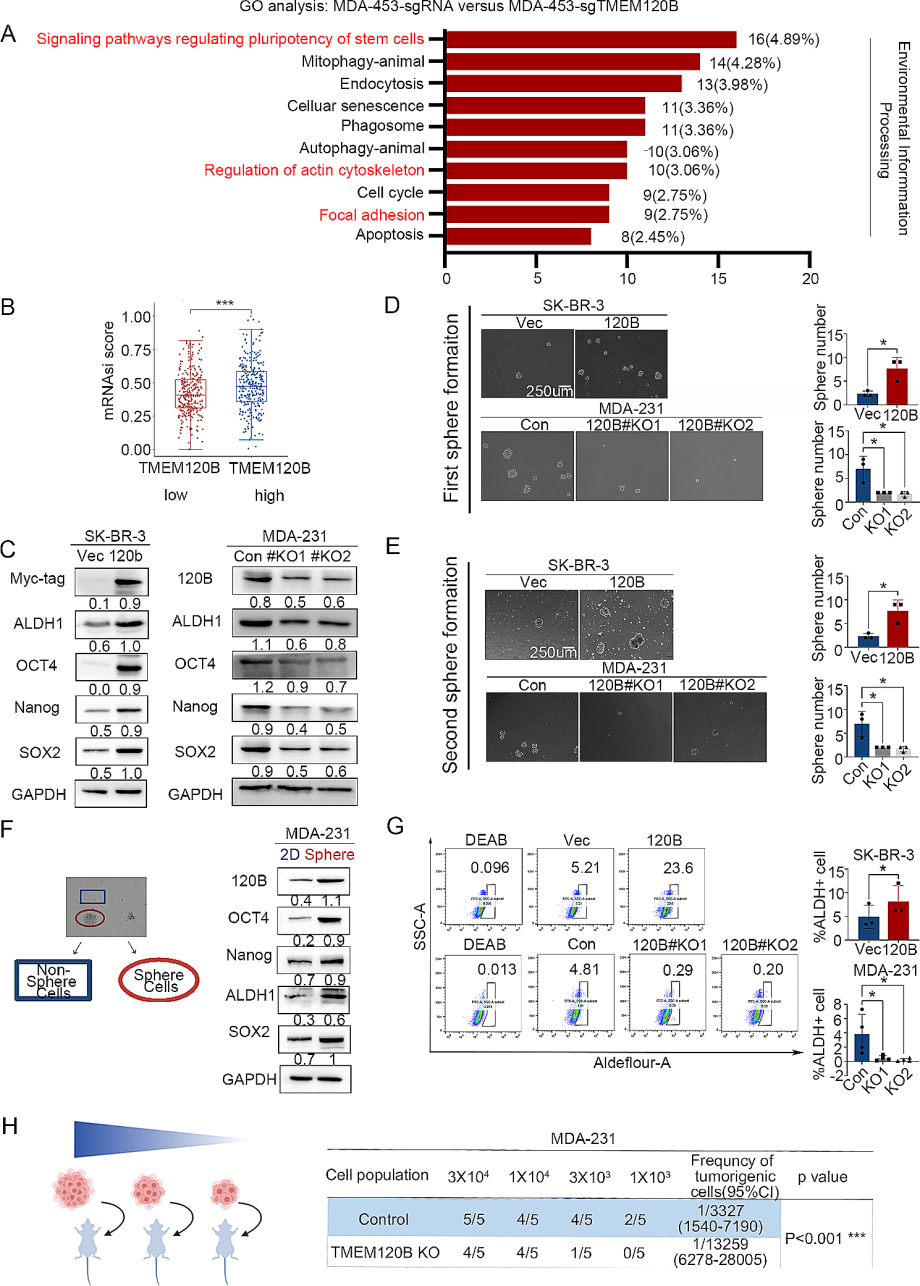
Overexpression of TMEM120B enhanced stemness of breast cancer cells. (**A**) GO analysis was performed to detect the biological process significantly correlated with the deletion of TMEM120B in MDA-453 cells. (**B**) Bioinformatics analysis for the mRNAsi Stemness score of TMEM120B (**C**) Immunoblotting of Myc-tag, ALDH1, OCT4, NANOG, SOX2, and GAPDH after overexpressing or deleting TMEM120B in SK-BR-3 and MDA-231 cells. Both the first (**D**, scale bar = 250 μm) and second round of sphere formation assays (**E**) were performed to examine the effects on stemness of cells after overexpressing or knocking out TMEM120B in SK-BR-3 or MDA-231 cells. (**F**) Immunoblotting of Myc-tag, OCT4, NANOG, ALDH1, SOX2, and GAPDH in MDA-231 cells in both non-sphere and sphere groups. (**G**) Flow cytometry assay detected the ratio of ALDH1^+^ cells upon overexpression or deletion of TMEM120B in SK-BR-3 or MDA-231 cells (H) Diluted injection xenograft assays to explore the effects on stemness of breast cancer cells upon depleting TMEM120B in MDA-231 cells *in vivo.* Quantification data are expressed as mean ± SD of three independent experiments (t-test, two-sided, *P < 0.05, **P < 0.01, ***P < 0.001)

Next, we explored the mechanism by which TMEM120B promoted breast cancer cell proliferation, invasion, and metastasis. RNA-sequencing was performed to identify the differentially expressed genes (DEGs) after deleting TMEM120B in MDA-453 cells, revealing 793 DEGs (fold change > 1.5), among which 687 genes were downregulated, and 106 genes were upregulated (PRJNA938979, https://www.ncbi.nlm.nih.gov/bioproject/PRJNA938979/; Additioanl file 4). Gene ontology (GO) analysis indicated that the DEGs were enriched in the following processes: signaling pathways regulating the pluripotency of stem cells, cell cycle, regulation of actin cytoskeleton, and focal adhesion (Fig. 3A). Previous studies have demonstrated that enhancing the stemness of breast cancer cells may accelerate their proliferation, invasion, and metastasis [47]. Interestingly, bioinformatics analysis indicated that TMEM120B expression was significantly positively correlated with breast cancer stemness (Fig. 3B). Western blotting indicated that the expression of pivotal breast CSC markers, such as ALDH1, OCT4, Nanog, and Sox2, was increased or decreased upon TMEM120B overexpression or inhibition (Fig. 3C and Additional file 3: Fig. S3A). Both the first and second rounds of sphere formation assays suggested that the stemness of breast cancer cells was enhanced or abrogated after the overexpression or depletion of TMEM120B in MCF-7 and SK-BR-3 or MDA-231 and MDA-453 cells (Fig. 3D-E and Additional file 3: Fig. S3B-C), respectively. Moreover, we isolated both sphere and adhesive MDA-231 cells and detected the expression of stem cell markers, which indicated that TMEM120B and stem cell markers were elevated in sphere cells compared with those in adhesive cells (Fig. 3F). Flow cytometry also indicated that the ratio of ALDH1-positive cells increased or decreased upon the overexpression or deletion of TMEM120B in SK-BR-3 or MDA-231 cells, respectively (Fig. 3G). Further, we assessed the effects of limiting dilution xenograft on breast cancer stemness upon TMEM120B depletion in MDA-MB-231 cells in vivo, which also indicated that overexpression of TMEM120B may strengthen breast cancer stemness (Fig. 3H).

### Overexpression of TMEM120B strengthened breast cancer stemness via TAZ-mTOR signaling axis

**Fig. 4:**
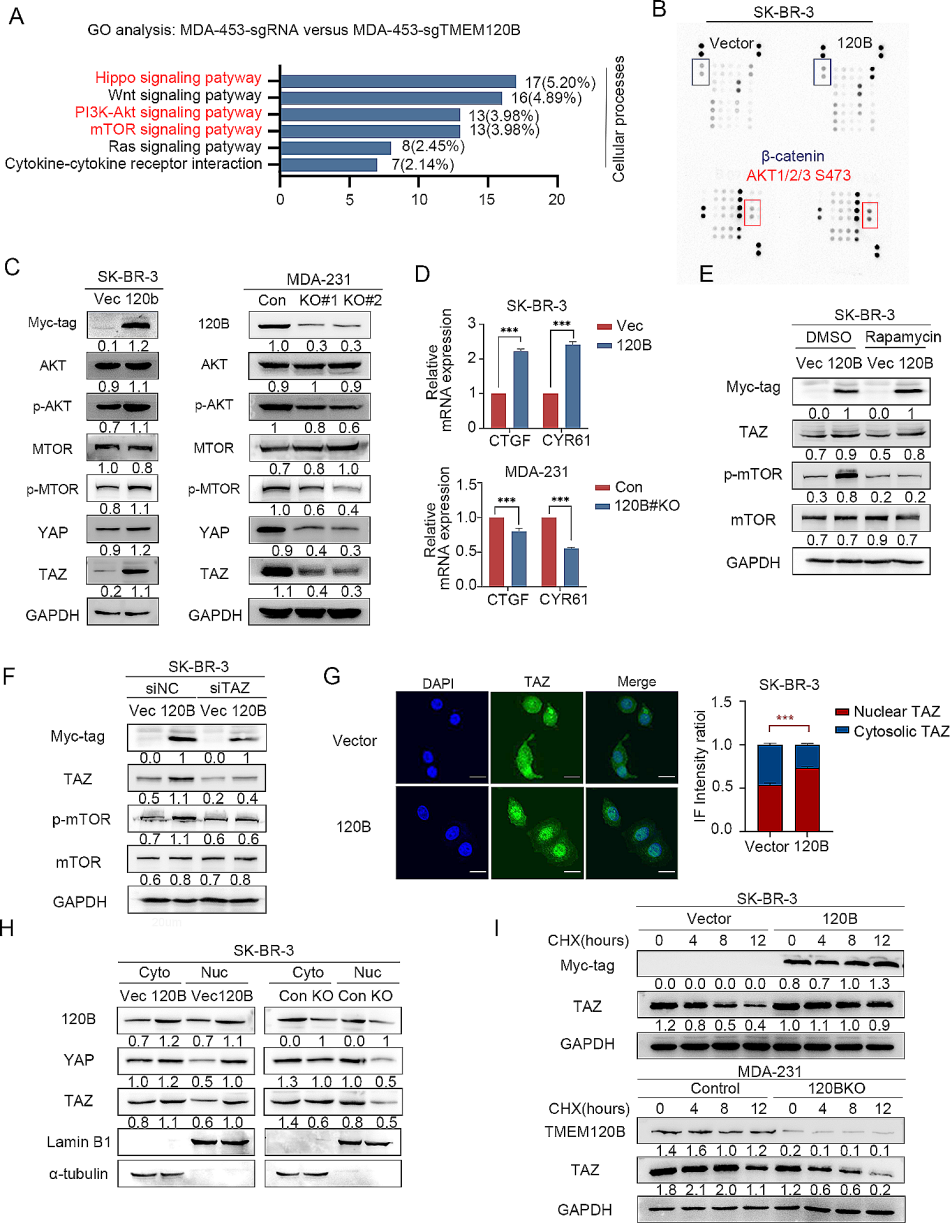
Overexpressing TMEM120 accelerated breast cancer stemness by activating TAZ-mTOR signaling axis. (**A**) KEGG analysis was conducted to detect the signaling pathway significantly correlated with deletion of TMEM120B in MDA-453 cells. (**B**) Phosphorylation antibodies array kit was used to explore the key signaling pathway involved in TMEM120B overexpression in SK-BR-3 cells. (**C**) Immunoblotting of Myc-tag, TMEM120B, AKT, p-AKT, mTOR, p-mTOR, YAP, TAZ, and GAPDH after overexpressing or silencing TMEM120B in SK-BR-3 or MDA-231 cells. (**D**) qPCR assay was used to investigate the alteration of the target genes of YAP/TAZ within ectopic or deleted TMEM120B in SK-BR-3 or MDA-231 cells. (**E**) Immunoblotting of Myc-tag, mTOR, p-mTOR, TAZ, and GAPDH after overexpressing TMEM120 with or without mTOR signaling pathway inhibitor rapamycin in SK-BR-3 cells. (**F**) Immunoblotting of Myc-tag, mTOR, p-mTOR, TAZ, and GAPDH after overexpressing TMEM120 with or without knocking down TAZ by siRNA in SK-BR-3 cells. Subcellular localization of TAZ was evaluated by immunofluorescence assay (**G**) or western blotting assay (**H**) within ectopic TMEM120B in SK-BR-3 cells. Scale bar = 20 μm. (**I**) After being treated with CHX at indicated time points, the expression of TAZ was evaluated by western blotting after overexpressing or silencing TMEM120B in SK-BR-3 or MDA-231 cells. Quantification data are expressed as mean ± SD of three independent experiments (t-test, two-sided, ***, *P* < 0.001)

We explored the signaling pathway transduction upon TMEM120B overexpression to maintain the stemness of breast cancer cells. Kyoto Encyclopedia of Genes and Genomes (KEGG) analysis of the differential genes from RNA-sequencing after deleting TMEM120B in MDA-453 cells indicated that Hippo, Wnt, AKT/mTOR signaling pathways were involved (Fig. 4A). Gene set enrichment analysis (GSEA) also indicated that mTOR signaling was positively correlated with higher TMEM120B expression (Additional file 3: Fig. S4A). We assessed the phosphorylation antibody array to screen the signaling pathways involved in TMEM120B overexpression, which indicated that the phosphorylation of AKT at serine 473 was increased; however, the phosphorylation of β-catenin, which is crucial for Wnt signal transduction, and other signaling pathways, was not altered (Fig. 4B). Hippo and PI3K-AKT signaling were chosen for further studies as they are both proven to be crucial in maintaining stemness of breast cancer cells [48–50].Subsequent western blotting indicated that levels of phosphorylated AKT, phosphorylated mTOR, and TAZ were significantly increased or decreased, and YAP level was slightly increased or decreased after the overexpression or depletion of TMEM120B in SK-BR-3 or MDA-231 cells, respectively (Fig. 4C). Additionally, qPCR was used to examine the expression of *CYR61* and *CTGF*, the downstream target genes of YAP and TAZ, and we found that *CYR61* and *CTGF* were upregulated or downregulated upon TMEM120B overexpression or deletion in SK-BR-3 or MDA-231 cells, respectively (Fig. 4D). Previous studies have demonstrated that both Hippo and AKT/mTOR signaling pathways are involved in maintaining breast cancer stemness [51–53]. Next, we assessed the specific mTOR inhibitor rapamycin or silenced TAZ with siRNA to clarify whether the enhancement of breast cancer stemness induced by overexpressing TMEM120B was dependent on the activation of both signaling pathways.Western blotting results revealed that adding 2 nmol rapamycin for 48 h or inhibiting TAZ may counteract the increase in the proliferation, invasion, and stemness of SK-BR-3 cells induced by TMEM120B overexpression (Additional file 3: Fig. S4B-G). Moreover, inhibiting mTOR signaling did not block the increase in TAZ level, whereas silencing TAZ using siRNA neutralized the elevation of phosphorylated mTOR (Fig. 4E-F), indicating that TAZ may act upstream of mTOR upon overexpression of TMEM120B.

We investigated the mechanism by which TMEM120B overexpression activates TAZ. The qPCR results indicated that overexpression or silencing of TMEM120B in SK-BR-3 or MDA-231 cells did not alter the mRNA expression of *TAZ* (Additional file 3: Fig. S4H). However, both immunofluorescence staining and western blotting indicated that nuclear TAZ expression increased or decreased after the overexpression or deletion of TMEM120B, respectively (Fig. 4G-H). Translocation of TAZ controls its stability, which is crucial for Hippo signaling pathway transduction [18]. We used cycloheximide (CHX) to block de novo protein synthesis and investigated the effect of TMEM120B overexpression on the stability of TAZ. The results revealed that TAZ degradation was blocked or accelerated upon TMEM120B overexpression or deletion in SK-BR-3 or MDA-231 cells, respectively (Fig. 4I).

### TMEM120B directly bound to MYH9 through their coil-coil domains and maintained breast cancer stemness

**Fig. 5:**
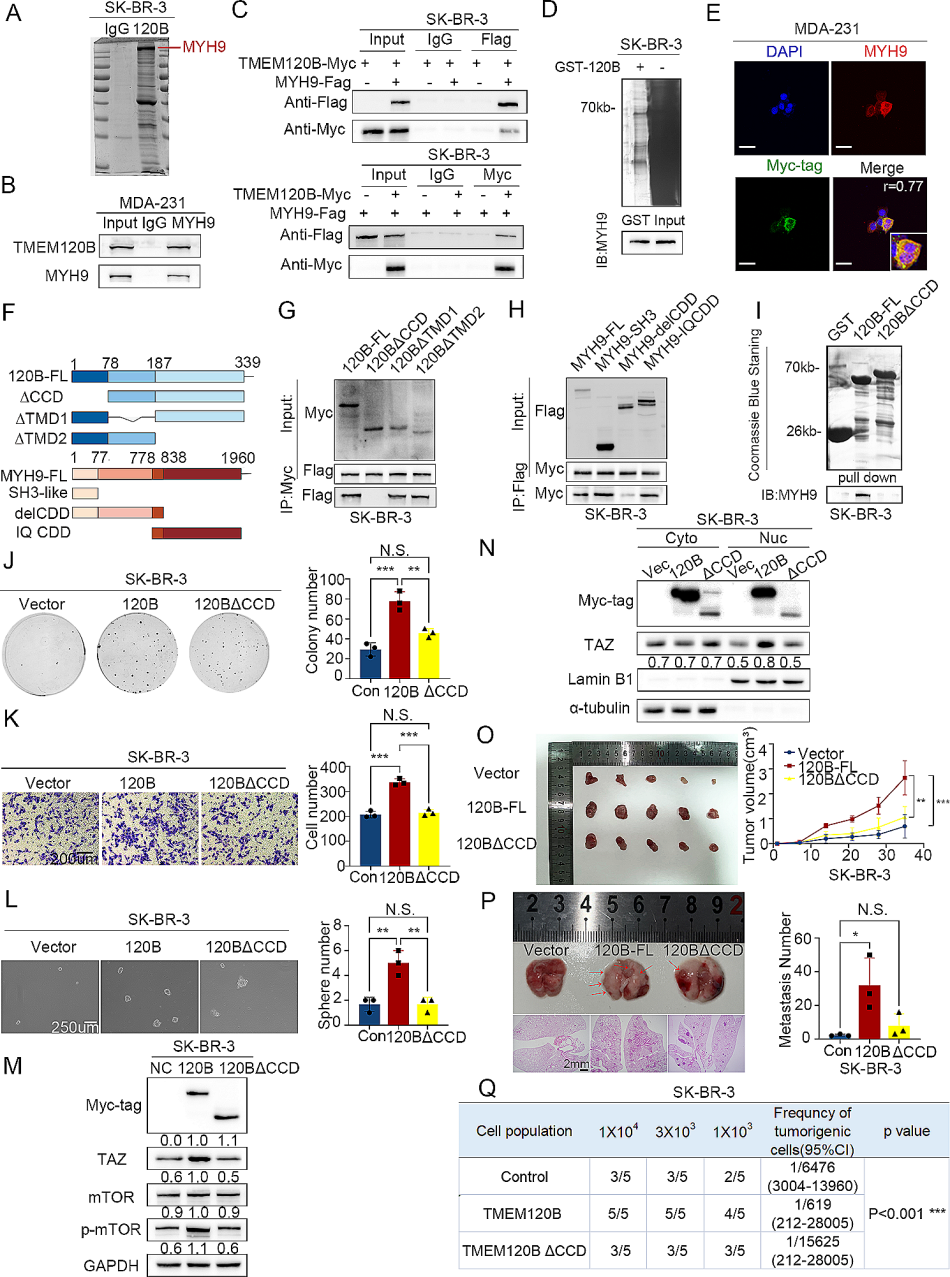
TMEM120B promoted breast cancer cell stemness by binding with MYH9 via their coil-coil domains. (**A**) Mass spectrometry (MS) analysis was performed to identify candidates for interaction with TMEM120B after overexpressing TMEM120B in SK-BR-3 cells. Endogenous (**B**) and exogenous co-IP assay (**C**) were assessed to detect the interaction between MYH9 and TMEM120B in MDA-231 and SK-BR-3 cells. (**D**) GST pull-down assay was used to confirm the direct binding between MYH9 and TMEM120B in SK-BR-3 cells. (**E**) Immunofluorescence assay was used to show the co-localization of TMEM120B and MYH9 in SK-BR-3 cells, subcellular location coefficient of TMEM120B–MYH9 interaction was quantified by Fiji software (scale bar = 50 μm) (**F**) Divergent TMEM120B and MYH9 splicing mutant plasmids were designed to examine the domain responsible for the interaction between TMEM120B and MYH9.(**I**) GST pull-down assay was performed to confirm the direct interaction between MYH9 and TMEM120B after overexpressing TMEM120B-WT or TMEM120B-∆CCD plasmids in SK-BR-3 cells. Colony formation assay (**J**), Transwell assay (**K**), and sphere formation assay (**L**) were performed to detect the effects on the proliferation, invasion, and stemness of breast cancer cells after transfecting TMEM120B, TMEM120B-∆CCD, and control plasmid in SK-BR-3 cells. (**M**) Immunoblotting of Myc-tag, mTOR, p-mTOR, TAZ, and GAPDH after overexpressing TMEM120B or TMEM120B-∆CCD in SK-BR-3 cells. (**N**) Immunoblotting was used to evaluate the expression of cytosolic or nuclear TAZ after overexpressing TMEM120B or TMEM120B-∆CCD in SK-BR-3 cells. The effects on proliferation, metastasis, and stemness were verified in nude mice by subcutaneous tumorigenesis (**O**), tail vein injection (**P**), and diluted injection xenografts assays (**Q**) by overexpressing TMEM120B and TMEM120B-∆CCD in SK-BR-3 cells. Quantification data are expressed as mean ± SD of three independent experiments (t-test, two-sided, **, *P* < 0.01, ***, *P* < 0.001)

An endogenous co-IP assay indicated that TMEM120B did not interact with TAZ, which revealed that TAZ stabilization by overexpression may not result from direct interactions (Additional file 3: Fig. S5A). Mass spectrometry (MS) was performed to identify candidates for interaction with TMEM120B. We found 316 potential binding proteins, as shown in Fig. 5A and Additional file 5. MYH9 and ACTN4 were used to test their interactions with TMEM120B, as they were in the top 10. Moreover, both are involved in modulating the cell actin cytoskeleton, which tightly modulates TAZ by affecting mechanical force [54, 55]. An endogenous co-IP assay indicated that MYH9, rather than ACTN4, interacts with TMEM120B in MDA-231 cells (Fig. 5B and Additional file 3: Fig. S5B). Subsequent co-IP revealed that exogenous TMEM120B interacted with MYH9 in SK-BR-3 cells (Fig. 5C). The GST pull-down assay showed that TMEM120B directly binds to MYH9 (Fig. 5D). Immunofluorescence assays suggested that TMEM120B co-localized with MYH9 in the cytoplasm of MDA-231 cells, which was quantified using the ImageJ software (Fig. 5E, *R* = 0.77). Next, we tested whether enhanced stemness induced by TMEM120B overexpression was dependent on MYH9. We transfected TMEM120B plasmids and MYH9-siRNA in SK-BR-3 cells; western blotting results indicated that elevated TAZ, phosphorylated mTOR, and ALDH1 were blocked by MYH9 silencing (Additional file 3: Fig. S5C). Additionally, MYH9 inhibition suppressed SK-BR-3 cells proliferation, invasion, and stemness(Fig. S5D-F). Divergent TMEM120B and MYH9 splice-mutant plasmids were designed to determine the exact domain responsible for the interaction between TMEM120B and MYH9 (Fig. 5F). The co-IP assay results suggested that the coil-coil domains in both TMEM120B and MYH9 dominated their binding in SK-BR-3 cells (Fig. 5G-H). GST pull-down assays also revealed that the coil-coil domain was crucial for TMEM120B–MYH9 binding (Fig. 5I). Overexpression of TMEM120B-∆CCD may neutralize the elevation of TAZ, phosphorylated mTOR, and the enhancement of proliferation, invasion, and stemness of SK-BR-3 cells both in vitro and in vivo (Fig. 5J-Q).

### TMEM120B stabilized MYH9 by preventing its ubiquitination to CUL9 in a competitive manner

**Fig. 6:**
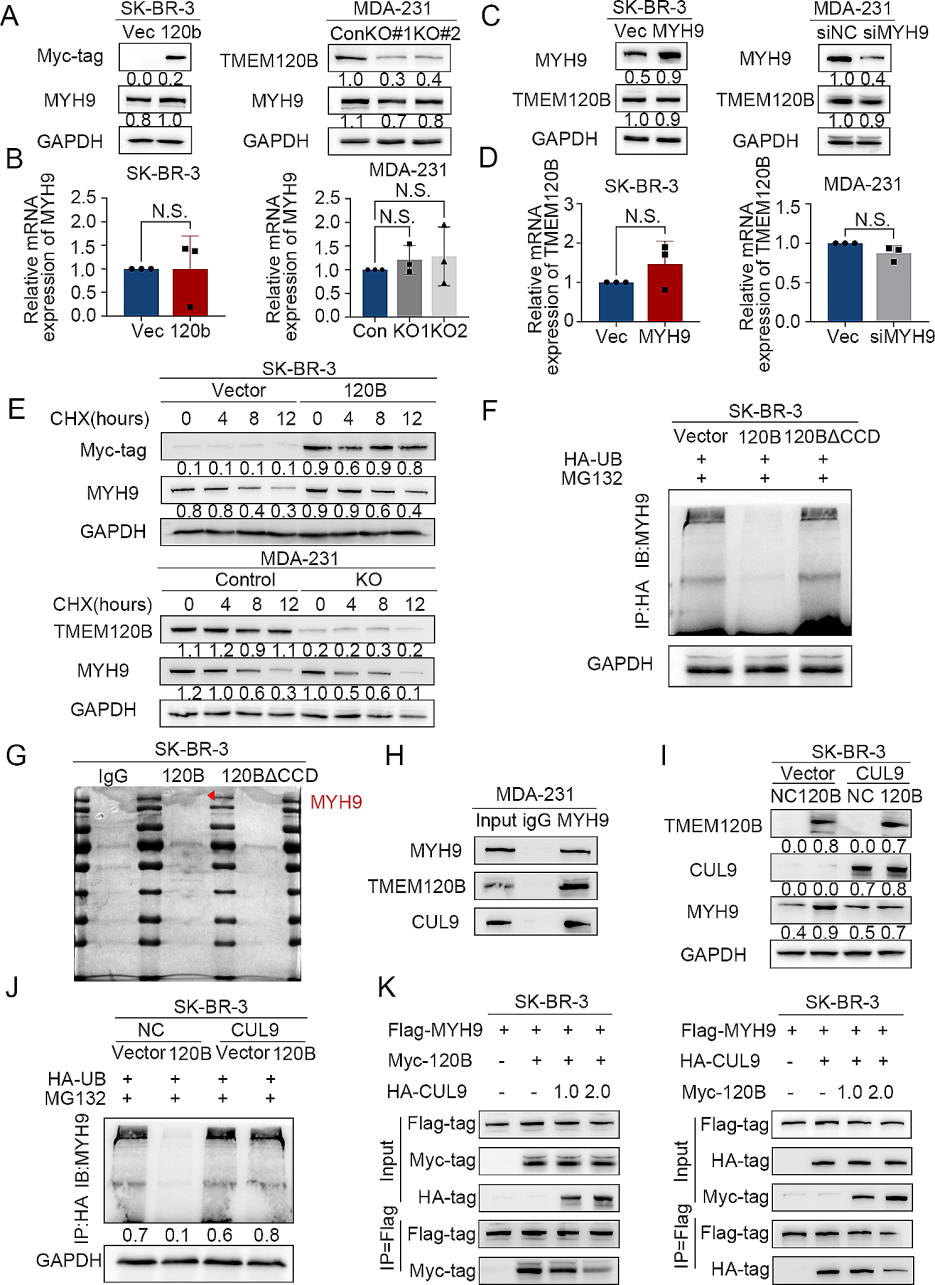
TMEM120B stabilized MYH9 by preventing its ubiquitin-mediated degradation from CUL9. (**A** and **B**) Western blotting and qPCR assays were performed to determine the protein and mRNA expression of MYH9, respectively, after overexpressing or deleting TMEM120B in SK-BR-3 or MDA-231 cells. (**C**-**D**) Western blotting and qPCR assays were performed to assess the protein and mRNA expression of TMEM120B, respectively, after overexpressing or deleting MYH9 in SK-BR-3 or MDA-231 cells. (**E**) After being treated with CHX at indicated time points, the expression of MYH9 was evaluated by western blotting after overexpressing or silencing TMEM120B in SK-BR-3 or MDA-231 cells. (**F**) The ubiquitination level of MYH9 was detected using western blotting after being transfected with TMEM120B, TMEM120B-∆CCD, and control plasmids in SK-BR-3 cells. (**G**) Mass spectrometry (MS) analysis was performed to identify candidates for interaction with ectopic TMEM120B or TMEM120B-∆CCD in SK-BR-3 cells, respectively. (**H**) Endogenous co-IP assay was performed to detect the interaction between MYH9, CUL9, and TMEM120B in MDA-231 cells. (**I** and **J**) Protein levels of MYH9 and the ubiquitination level were assessed using western blotting after transfecting TMEM120B alone or co-transfecting both TMEM120B and CUL9 in SK-BR-3 cells. (**K**) Co-IP assay was used to evaluate the interaction among TMEM120B, MYH9, and CUL9 after overexpressing TMEM120B and CUL9 in different doses in SK-BR-3 cells. Quantification data are expressed as mean ± SD of three independent experiments (t-test, two-sided)

MYH9 protein levels, rather than mRNA levels, were significantly increased or decreased when TMEM120B was overexpressed or deleted in SK-BR-3 or MDA-231 cells, respectively (Fig. 6A-B). However, the mRNA and protein levels of TMEM120B were not altered by MYH9 overexpression or silencing in SK-BR-3 or MDA-231 cells (Fig. 6C-D). CHX was used to block the de novo protein synthesis of MYH9, which revealed that overexpression or deletion of TMEM120B may abolish or enhance the degradation of MYH9 in SK-BR-3 cells or MDA-231 cells (Fig. 6E). MYH9 is unstable and can be degraded via proteasome-dependent ubiquitination [35]. Our results, which are consistent with those of previous studies, indicated that MYH9 ubiquitination was abrogated or enhanced after the overexpression or depletion of TMEM120B in SK-BR-3 or MDA-231 cells, respectively (Fig. 6F and Additional file 3: Fig. S6A-B). Intriguingly, overexpressing TMEM120B-∆CCD may counteract the decreased ubiquitination induced by overexpressing TMEM120B in SK-BR-3 cells (Fig. 6F). TMEM120B has not been reported to function as an E3-ubiquitin ligase, which indicated that the TMEM120B–MYH9 interaction may prevent the degradation of MYH9 by a certain E3-ubiquitin ligase. MS analysis was performed to identify the candidate E3-ubiquitin ligases after overexpressing TMEM120B and TMEM120B-∆CCD in SK-BR-3 cells (Fig. 6G). Among the 141 proteins screened in the TMEM120B group (but not in the TMEM120B-∆CCD group), we identified CUL9, an E3-ubiquitin ligase and E3-adaptor proteins RACK1 and UBD protein BRSK2 (Additional file 6). Interestingly, MYH9 remained on the candidates list, confirming our previous co-IP results. Co-IP assays indicated that TMEM120B, MYH9, and CUL9 formed ternary complexes in MDA-231 cells (Fig. 6H). Overexpression of TMEM120B, together with CUL9, abolished the elevation of MYH9 and the reduction in MYH9 ubiquitination (Fig. 6I and J). Overexpression of TMEM120B and CUL9 may counteract the elevated proliferation, invasion, and stemness induced by TMEM120B overexpression in SK-BR-3 cells (Additional file 3: Fig. S6C-E). Further, the interaction between TMEM120B and MYH9 was blocked by CUL9 overexpression in a dose-dependent manner, indicating that TMEM120B competitively binds to MYH9 from CUL9, thus preventing the degradation of MYH9 (Fig. 6K).

### TMEM120B–MYH9 interaction enhanced breast cancer stemness via β1-integrin-FAK-TAZ-mTOR axis

**Fig. 7:**
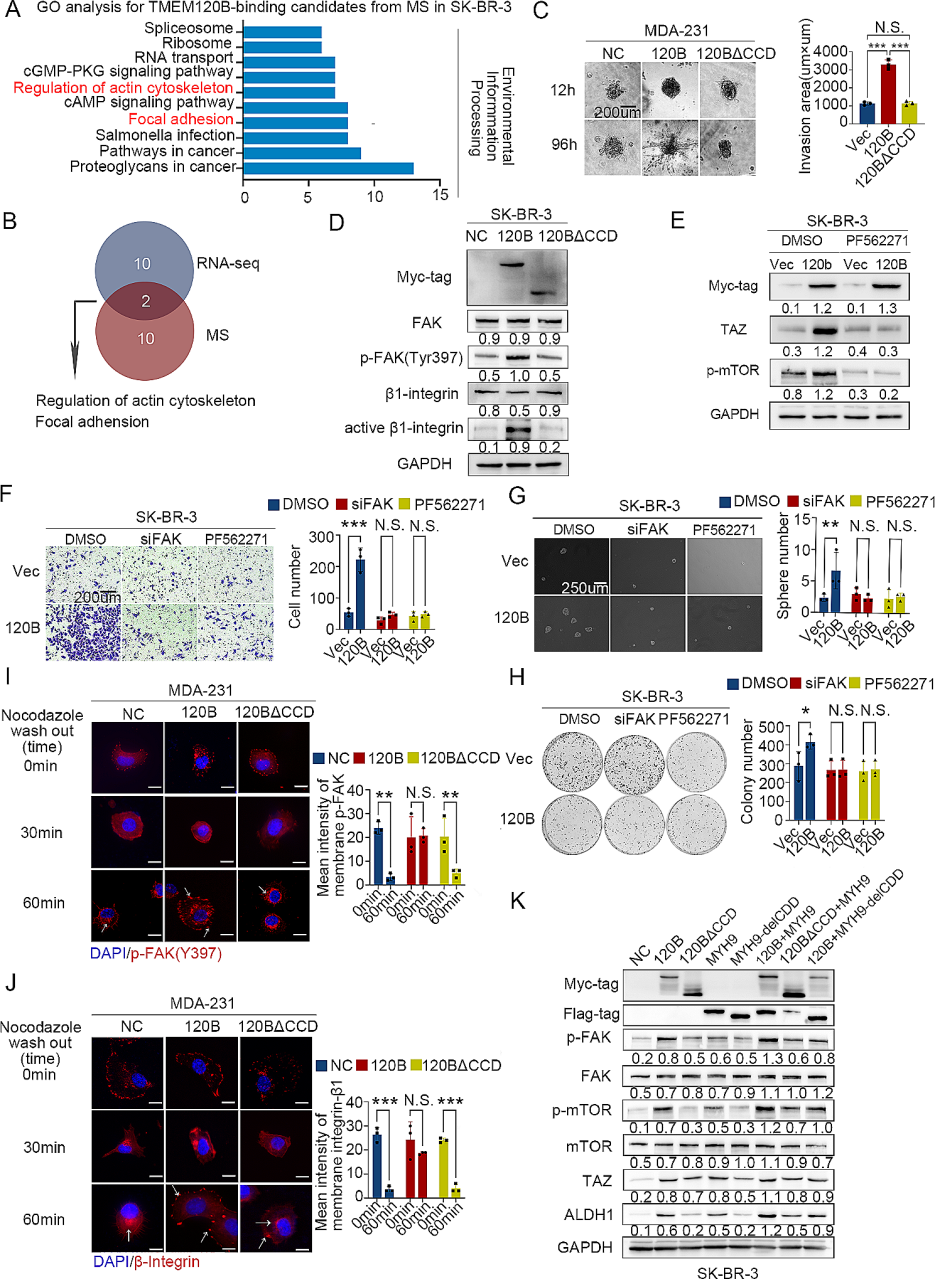
TMEM120B–MYH9 interaction activated the TAZ-mTOR axis by accelerating FAK assembly. (**A**) GO analysis for TMEM120B interaction candidates from MS analysis after overexpressing TMEM120B in SK-BR-3 cells. (**B**) Venn analysis for the overlap between RNA-seq and MS analysis. (**C**) 3D collagen gel invasion assay was performed after overexpressing TMEM120B or TMEM120B-∆CCD and control in MDA-231 cells. (**D**) Immunoblotting assay was performed to evaluate the expression of Myc-tag, FAK, p-FAK (Tyr397), β1-integrin, active-β1-integrin, and LaminB1 after transfecting TMEM120B-myc, TMEM120B-∆CCD-myc, and control plasmids in SK-BR-3 cells. (**E**) Immunoblotting of Myc-tag, p-mTOR, TAZ, and GAPDH after overexpressing TMEM120B with or without FAK signaling pathway inhibitor PF562271 in SK-BR-3 cells. Transwell assay (**F**), sphere formation assay (**G**), and colony formation assay (**H**) were performed to detect the effect on the invasion, stemness, and proliferation of breast cancer cells upon ectopic TMEM120B or ∆TMEM120B-CCD in SK-BR-3 cells. Representative immunofluorescence images of p-FAK (**I**) and β1-integrin (**J**) after treatment with nocodazole (NZ), followed by washout for 0, 30, and 60 min. Scale bar = 10 μm. (**K**) Immunoblotting of Myc-tag, Flag-tag, FAK, p-FAK(Tyr397), mTOR, p-mTOR, TAZ, ALDH1, and GAPDH after transfected with TMEM120B-myc, TMEM120B-∆CCD-myc, MYH9-flag, MYH9-delCCD-flag alone, or TMEM120B-myc + MYH9-flag, TMEM120B-∆CCD-myc + MYH9-flag, or TMEM120B-myc + MYH9-delCCD-flag in SK-BR-3 cells, respectively. Quantification data are expressed as mean ± SD of three independent experiments (t-test, two-sided, *P < 0.05, **P < 0.01, ***P < 0.001)

MYH9 is responsible for the formation of FAs, thereby promoting colon cancer progression [35]. We performed GO analysis of TMEM120B interaction candidates from the MS analysis in SK-BR-3 cells and found that they were enriched in the modulation of the cell skeleton and focal adhesion (Fig. 7A), which was consistent with the RNA-seq results (Fig. 7B). Tang et al. showed that the integrin-FAK signaling axis is involved in mechanical force transduction and accelerates the nuclear translocation of YAP/TAZ [26]. First, a 3D collagen gel invasion assay was performed to evaluate whether TMEM120B is involved in mechanical force transduction. The results showed that overexpression of TMEM120B, rather than TMEM120B-∆CCD, enhanced 3D invasion in MDA-231 cells (Fig. 7C). Western blotting assay indicated that overexpression of TMEM120B increased phosphorylated FAK levels in Tyr 397 and active-β1-integrin; however, ectopic TMEM120B-∆CCD did not (Fig. 7D). PF562271, a FAK signaling-specific inhibitor, was applied after overexpressing TMEM120B in SK-BR-3 cells, and western blotting results revealed that the elevation of TAZ and phosphorylated mTOR was blocked (Fig. 7E). SK-BR-3 cells proliferation, invasion, and stemness were abrogated by FAK inhibition (Fig. 7F-H). FA assembly can be modulated by recycling integrins from the membrane to the cytosol upon activation of mechanical force signaling [56]. FA assembly was evaluated following NZ treatment. After treatment with NZ for 30 min, no obvious membranous integrin or phosphorylated FAK was observed (Fig. 7I and J). However, overexpression of TMEM120B, rather than TMEM120B-∆CCD in MDA-231 cells, accelerated membrane-expressed β1-integrin and phosphorylated FAK after treatment with NZ for 60 min (Fig. 7I and J). Finally, we overexpressed TMEM120B, TMEM120B-∆CCD, MYH9, MYH9-∆CCD alone or co-transfected TMEM120B + MYH9, TMEM120B-∆CCD + MYH9, and TMEM120B + MYH9-∆CCD into breast cancer cells. Western blotting results indicated that co-transfected TMEM120B + MYH9, rather than TMEM120B-∆CCD + MYH9 or TMEM120B + MYH9-∆CCD, significantly increased the expression of phosphorylated FAK and mTOR, TAZ, and ALDH1 (Fig. 7K). Flow cytometry also indicated that the ratio of ALDH1-positive cells elevated more than the other groups upon co-transfecting TMEM120B + MYH9 (Additional file 3: Fig. S6F).Our results indicated that the TMEM120B–MYH9 interaction may enhance breast cancer stemness by activating the β1-integrin-FAK-TAZ-mTOR signaling axis.

### Overexpression of TMEM120B promoted docetaxel and doxorubicin therapy resistance

**Fig. 8:**
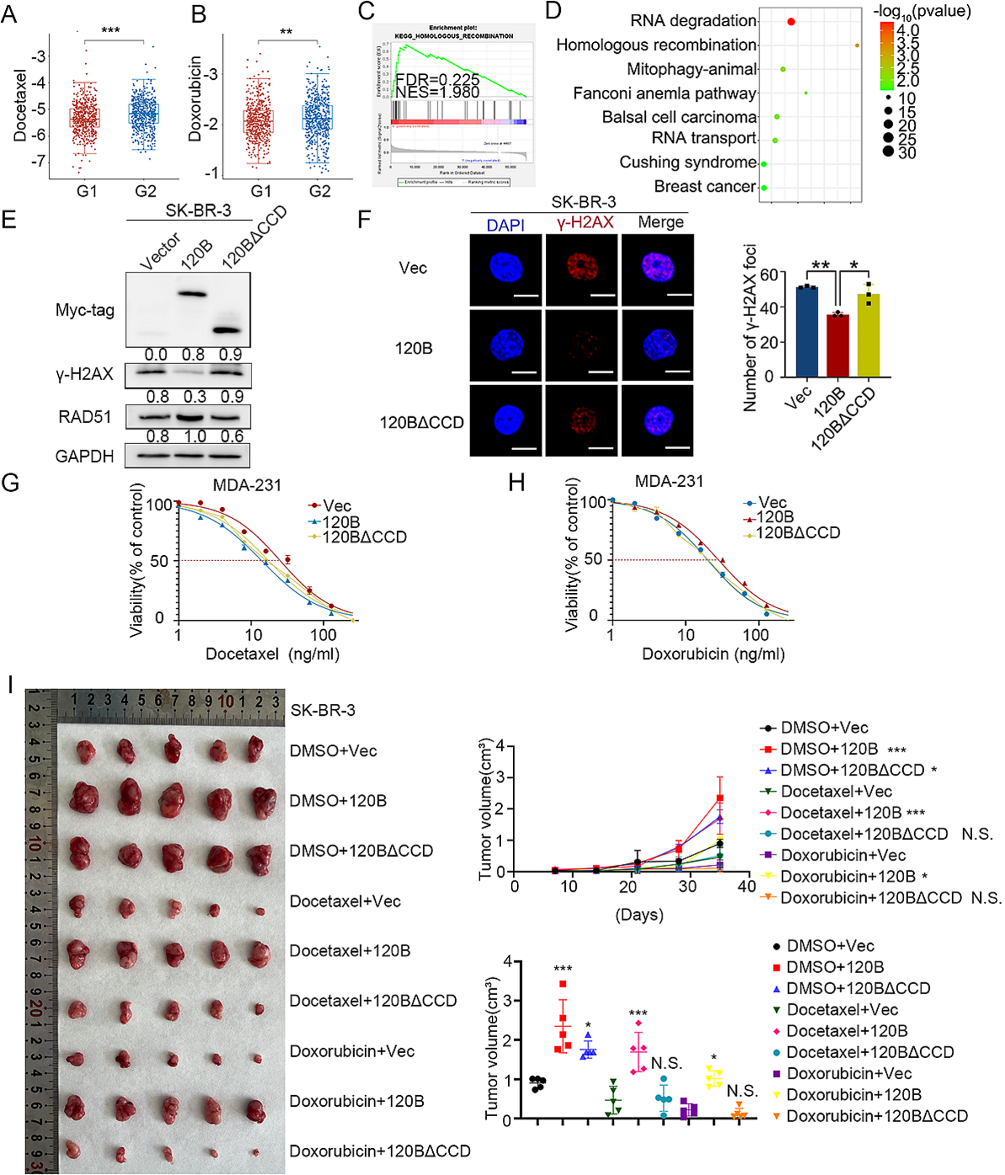
Overexpression of TMEM120B promoted chemotherapy resistance both in vitro and in vivo. The TCGA database was used to examine the relationship between TMEM120B expression and the effect of chemotherapy with docetaxel (**A**) and doxorubicin (**B**). GSEA from TCGA database (**C**) and RNA-seq by deleting TMEM120B in MDA-453 cells (**D**). (**E**) The expression of Myc-tag, RAD51, γ-H2AX, and GAPDH was evaluated by western blotting after overexpressing TMEM120B or TMEM120B-∆CCD and control in SK-BR-3 cells. (**F**) Representative immunofluorescence images of the foci number of γ-H2AX after overexpressing TMEM120B or TMEM120B-∆CCD and control in SK-BR-3 cells (scale = 10 μm). IC50 values in SK-BR-3 cells overexpressing TMEM120B after treatment with docetaxel (**G**) or docetaxel (**H**). (**I**) Xenografts assays were assessed after overexpressing TMEM120B or TMEM120B-∆CCD in SK-BR-3 cells with docetaxel or docetaxel. Quantification data are expressed as mean ± SD of three independent experiments (t-test, two-sided, *P < 0.05, **P < 0.01, ***P < 0.001)

CSC expansion is a major cause of therapeutic resistance [5, 57]. Based on the TCGA database, we found that *TMEM120B* mRNA levels were significantly higher in the docetaxel- and doxorubicin-resistant groups than in the docetaxel- and doxorubicin-sensitive groups (Fig. 8A-B). Both GSEA and RNA-seq results revealed that TMEM120B may be involved in homologous recombination (HR) (Fig. 8C-D). Subsequent western blotting indicated that the level of RAD51, an HR marker, was increased and that of γ-H2AX, a DNA damage marker, was suppressed upon TMEM120B overexpression rather than TMEM120B-∆CCD overexpression (Fig. 8E). The immunofluorescence assay also indicated that the nuclear foci of γ-H2AX were significantly abrogated within ectopic TMEM120B, but not in TMEM120B-∆CCD in SK-BR-3 cells (Fig. 8F). We treated MDA-231 cells with varying concentrations of docetaxel and doxorubicin and measured IC50. The results suggested that overexpressing TMEM120B increased IC50 values substantially (docetaxel, 25.27 ng/mL; doxorubicin, 28.23 ng/mL); however, there was no considerable alteration between SK-BR-3 cells overexpressing TMEM120B-∆CCD (docetaxel, 17.52 ng/mL, doxorubicin, 20.15 ng/mL) and the negative control (docetaxel, 14.06 ng/mL; doxorubicin, 20.07 ng/mL; Fig. 8G-H). Additionally, xenograft assays suggested that TMEM120B overexpression, rather than TMEM120B-∆CCD overexpression in SK-BR-3 cells, accelerated docetaxel and doxorubicin treatment resistance in vivo (Fig. 8I).

### TMEM120B expression positively correlated with neoadjuvant chemotherapy resistance

**Fig. 9:**
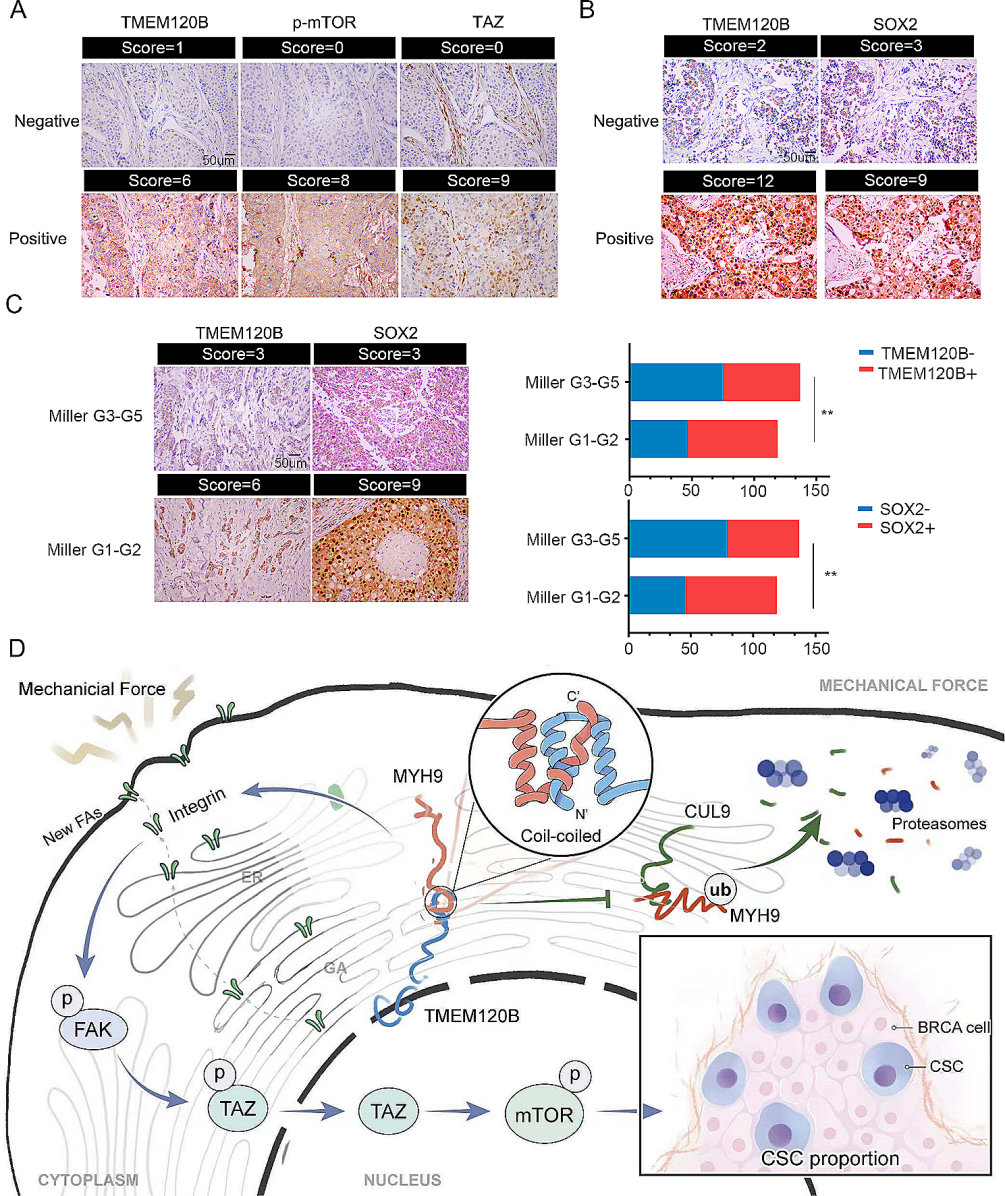
TMEM120 expression positively correlated with TAZ, p-mTOR, SOX2, and chemotherapy resistance in breast cancer specimens. (**A**) Representative images of immunohistochemistry staining of TMEM120B, phosphorylated mTOR, and TAZ in human breast cancer specimens. (**B**) Representative images of immunohistochemistry staining of TMEM120B and SOX2 human breast cancer specimens. (**C**) Representative images of immunohistochemistry staining of TMEM120B in specimens from breast cancer patients with varying therapeutic effects evaluated using Miller/Payne Grades after neoadjuvant chemotherapy. (**D**) Pathway diagram for TMEM120B functional activity interaction with MYH9 in breast cancer cells. Quantification data are expressed as mean ± SD of three independent experiments (t-test, two-sided, ***, *P* < 0.001)

IHC staining was performed to explore the correlation between TMEM120B expression and its downstream factors in human breast cancer samples. TMEM120B expression was significantly and positively correlated with phosphorylated mTOR (*P* < 0.001) and TAZ (*P* < 0.001; Fig. 9A; Table 4). SOX2 expression, which was assessed as a biomarker of breast CSCs, was also positively correlated with TMEM120B expression (*P* < 0.001; Fig. 9B; Table 5). Additionally, we assessed TMEM120B and SOX2 expression in specimens from patients with varying therapeutic effects, as evaluated by the Miller/Payne Grades after neoadjuvant chemotherapy. The IHC staining results indicated that TMEM120B and SOX2 expression in patients who were sensitive to treatment (Miller/Payne Grade 1–2) was significantly lower than that in patients who exhibited resistance (Miller/Payne Grade 3–5, *P* = 0.012 for TMEM120B, *P* = 0.003 for SOX2; Fig. 9C).Moreover, we had also checked TMEM120B expression who had not received neoadjuvant chemotherapy but only got chemotherapy treatment after surgery. We found that TMEM120B expression correlated with poor overall response rate (ORR) after receiving chemotherapy, which revealed that in the patients with positive TMEM120B expression, the ORR was 18.75%, whereas, in the patient with negative TMEM120B expression, the ORR was 56.5% (*P* = 0.071, Additional file 3: Fig.S7A).

**Table 4:** Correlation of TMEM120B with the expression of TAZ and mTOR

		TMEM120B		r	P
		Negative	Positive		
TAZ	Negative	15	6	0.618	<0.001
	Positive	3	24		
mTOR	Negative	14	6	0.567	<0.001
	Positive	4	24		

**Table 5:** Correlation of TMEM120B with the expression of SOX2

		TMEM120B		r	P
		Negative	Positive		
SOX2	Negative	15	7	0.618	<0.001
	Positive	4	24		

Our study revealed that TMEM120B was highly expressed in breast cancer and other malignant carcinomas, and its expression was significantly correlated with advanced TNM stage, positive lymph node metastasis, and poor prognosis. Overexpression of TMEM120B may strengthen breast cancer stemness via the β1-integrin-FAK-TAZ-mTOR signaling axis by binding to MYH9. Moreover, TMEM120B stabilized MYH9 by preventing its degradation by CUL9 in a ubiquitin-dependent manner. Further, the overexpression of TMEM120B accelerated chemotherapy resistance (Fig. 9D).

## Discussion

Our study demonstrated that TMEM120B was highly expressed in the cytoplasm and was correlated with advanced TNM stage, lymph node metastasis, and poor prognosis. TMEM120B accelerated the cycling of β1-integrin and the assembling of FAs by activating the TAZ-mTOR signaling axis via binding to MYH9 through its coil-coil domain, thus strengthening proliferation and invasion, maintaining the stemness of breast cancer cells, and promoting chemoresistance. TMEM120B–MYH9 interaction also prevented the ubiquitination degradation of MYH9 by CUL9 in a competitive manner.

IHC staining indicated that TMEM120B was expressed mainly in the cytosol and fewly in the nucleus of breast cancer cells. TMEM120B is considered a nuclear lamina-related protein [37, 58]. However, our results suggested that TMEM120B is mainly localized in the cytoplasm of breast cancer cells rather than in the nuclear lamina.To further explore the biological roles of TMEM120B within diverse subcellular localization. First, we performed statistical analysis to seek the correlations between clinicopathlogic factors and TMEM120B within different localization, which revealed that only cytosolic TMEM120B significantly correlated with advanced TNM stage and positive lymph node metastasis rather than nuclear TMEM120B; Second, nuclear localization sequences (NLS) was predicted (http://nls-mapper.iab.keio.ac.jp/, Additional file 3: Fig.S8A). TMEM120B-Full length (FL) or TMEM120B-NLS-mut were transfected into SK-BR-3 cells, it revealed that TMEM120B-NLS-mutant was localized only in cytoplasm by IF staining (Additional file 3: Fig.S8B).Sphere formation, colony formation and transwell assay indicated that SK-BR-3 cells proliferation, invasion and stemness were not affect after overexpressing TMEM120B-NLS-mutant comparing with overexpressing TMEM120B-FL (Additional file 3: Fig. S8C-E). Subsequent western blotting assay also revealed no visible alteration by overexpressing TMEM120B-FL and TMEM120B-NLS-mutant in SK-BR-3 cells, respectively (Additional file 3: Fig. S8F).Third, TMEM120B promoted breast cancer cells by binding with MYH9 which was previously reported as a cytosolic protein [33, 34].Therefore, from our current results, cytosolic TMEM120B may serve as an oncogenic protein rather than nuclear one, however, the function of TMEM120B should be further investigated in the future.

RNA-sequencing data revealed that TMEM120 may be involved in modulating stemness and the Hippo, Wnt, PI3K-AKT, and mTOR signaling pathways. Subsequent phosphorylation antibody array assay also confirmed that overexpression of TMEM120B may elevate phosphorylation of AKT in serine 473; however, phosphorylation of β-catenin was not altered.We further select PI3K-AKT and Hippo signaling pathway for further investigation. AKT signaling pathway was chosen as both RNA-seq and phosohorylation antibody array indicated TMEM120B may be involved in modulating signaling transduction. Hippo signaling pathway was previously proven to be crucial for maintaining stemnes of breast cancer cells, moreover, the core effector YAP/TAZ are also demonstrated to be key nuclear tranductor in mechanicl force signaling activation [24, 25]. Regulation of actin cytoskeleton and focal adhesion were also idenfied from RNA-seq, which are crucial in promoting translocation of YAP/TAZ into nucleus upon mechanical force transduction [59, 60].However,in our study, overexpression of TMEM120B increased the expression of TAZ but not that of YAP. Our results, consistent with those of Michelangelo et al., indicate that TAZ may be crucial for maintaining the stemness of breast cancer cells [18]. Endogenous co-IP assays revealed that TMEM120B did not interact with TAZ, and subsequent MS assays indicated that TMEM120B bound to MYH9. Our findings revealed that the coil-coil domain in both proteins is crucial for their interaction. A previous study demonstrated that MYH9 promotes colon cancer progression by modulating FA assembly. Both RNA-seq and GO analyses of TMEM120B-binding proteins indicated that the cytoskeleton and FAs were involved. β1-integtrin-FAK-YAP/TAZ axis also accelerates the induction of mechanical force [20, 51]. Overexpressing of TMEM120B promoted breast cancer 3D invasion, which all indicated that TMEM120B were involved in modulating mechanical force transduction.β1-integrin cycling and FA assembly were enhanced in the TMEM120B–MYH9 interaction, thus promoted translocation of TAZ into nucleus as well as subsequent elevation of phosohorylation of mTOR.

Intriguingly, we found that overexpression of TMEM120B elevated the expression of MYH9, and ubiquitination of MYH9 was abrogated. A previous study indicated ATG9B–MYH9 interaction may prevent ubiquitination degradation [35]. However, in our MS assay, ATG9 was not considered a binding partner, and another E3 ubiquitin ligase, CUL9, was identified. Subsequent co-IP assays indicated that TMEM120B, MYH9, and CUL9 may form a ternary complex. Further, TMEM120B may prevent the ubiquitination and degradation of MYH9 in a competitive manner with CUL9.

Our study revealed that TMEM120B binds and stabilizes MYH9 via its coil-coil domains by preventing ubiquitination degradation of MYH9 induced by CUL9. The TMEM120B–MYH9 interaction may accelerate the β1-integrin/FAK-TAZ-mTOR signaling axis to maintain the stemness of breast cancer cells, thus promoting chemoresistance.

## Conclusion

Our study demonstrated that TMEM120B bound to and stabilized MYH9 by preventing its degradation by CUL9 in a competitive manner. This interaction activated the β1-integrin/FAK-TAZ-mTOR signaling axis, which maintains stemness and accelerates chemotherapy resistance.

### Electronic supplementary material

Below is the link to the electronic supplementary material.

Additional file 1: Supplementary Meterials and Methods

Additional file 2: Primers

Additional file 3: Supplementary Figure S1-S8

Additional file 4: DEGs within TMEM120B-KO cells

Additional file 5: Candidates for TMEM120B binding partner

Additional file 6: Candidates for TMEM120B and TMEM120B-delta CCD binding partner

## Data Availability

The data supporting the results of this study can be obtained from the corresponding author upon request.

## Abbreviations

TMEM120B: Transmembrane 120B
CSC: cancer stem cells
MST: Mammalian STE20-like protein kinase
LATS: large tumour suppressor kinase
ECM: Extracellular matrix
TAZ: transcriptional coactivator with PDZ-binding motif
qPCR: quantitative polymerase chain reaction
FAK: focal adhesion kinase
YAP: yes-associated protein
MYH9: Myosin heavy chain 9
IHC: immunohistochemistry
TCGA: The Cancer Genome Atlas
TNBC: triple-negative breast cancer
DEGs: differentially expressed genes
KEGG: kyoto encyclopedia of genes and genomes
CHX: cycloheximide
co-IP: co-immunoprecipitation
MS: Mass spectrometry
GO: gene ontology
CYR61: cysteine-rich angiogenic inducer 61
GSEA: gene set enrichment analysis
CTGF: connective tissue growth factor
ORR: overall response rate
