# Supplementary material for: TMEM120B strengthens breast cancer cell stemness and accelerates chemotherapy resistance via β1-integrin/FAK-TAZ-mTOR signaling axis by binding to MYH9
Source: Breast Cancer Res. 2024 Mar 19;26:48. doi: 10.1186/s13058-024-01802-z (PMC10949598; doi:10.1186/s13058-024-01802-z)
Supplement: Supplementary file 1 — Additional file 1: Supplementary Meterials and Methods [file 13058_2024_1802_MOESM1_ESM.docx]

**Patients and clinical specimens**

Primary tumor specimens were obtained from 140 patients with breast cancer, including 77 patients with triple-negative cancer and 63 patients with non-triple-negative tumors. All patients diagnosed with invasive ductal carcinoma (IDC) underwent complete surgical resection at the Affiliated Cancer Hospital of China Medical University between 2001 and 2003. Complete follow-up data (from 2003 to 2014) were available for all the 140 patients. Patient survival was defined as the time from the day of surgery to the end of the follow-up period or the date of death due to recurrence or metastasis. None of the patients had received radiotherapy or chemotherapy before undergoing surgical resection, and all patients were treated with routine chemotherapy after surgery. An additional 256 patients, who received neoadjuvant chemotherapy treatment from 2010 to 2021, were included. Of these, 20 patients were diagnosed as Luminal A subtype, 143 Luminal B, 63 Her-2 overexpressing and 30 TNBC.168 patients receive E (Epirubicin)+C (Cyclophosphamide) plans for 4 cycles followed by T (Docetaxel) for 4 cycles and 62 received TEC plans for 6 cycles, 26 patients received TCbH plans (Docetaxel+Carboplatin+Trastuzumab) for 6 cycles. 119 patients were classified as Miller–Payne (MP) grade 1 and 2, and the other 137 patients were MP grade 3–5. For breast cancer patients only receiving chemotherapy after surgery but not neoadjuvant chemotherapy, 4 patients were diagnosed as Luminal A subtype, 18 Luminal B, 15 Her-2 overexpressing and 9 TNBC. 22 patients receive E (Epirubicin)+C (Cyclophosphamide) plans for 4 cycles followed by T (Docetaxel) for 4 cycles and 9 received TEC plans for 6 cycles, 15 patients received TCbH plans (Docetaxel+Carboplatin+Trastuzumab) for 6 cycles. All patients repeated CT scan four weeks after finishing chemotherapy. Target lesions and tumor response were evaluated by Response Evaluation Criteria In Solid Tumors (RECIST) 1.1(1). Complete response (CR) and partial response (PR) were regarded as good efficacy, and stable disease (SD) and progressive disease (PD) were defined as poor outcomes in this study.

A total of 16 freshly isolated specimens, including both tumor tissue and the corresponding normal tissues, were stored at −70°C immediately after resection for subsequent protein extraction. Furthermore, to determine the universality of TMEM120B expression patterns in epithelial malignant tumors, we collected specimens from 20 cases of lung cancer, 29 cases of breast cancer, 21 cases of gastric cancer, 24 cases of colon cancer, 20 cases of ovarian cancer and relative non-cancerous normal tissues for immunohistochemical comparisons.

**Functional enrichment analyses**

Database for Annotation, Visualization, and Integrated Discovery (DAVID) (https://david.ncifcrf.gov/summary.jsp), an online tool for gene functional enrichment, was used for gene ontology (GO) analysis (cellular component, molecular function, and biological process) and Kyoto Encyclopedia of Genes and Genomes (KEGG) pathway analysis of the 729 differentially expressed genes (DEGs) shared between high and low expression of the TMEM120B group. The results were visualized using the ggplot2 R package. Statistical significance was set at P <0.05. Gene set enrichment analysis (GSEA) was adopted to identify the signaling pathways involved in the elevated TMEM120B-related gene signature in breast cancer patients. Statistical significance was set at p < 0.05. The pathways used for GSEA were obtained from the Molecular Signatures Database (MSigDB) (http://software.broadinstitute.org/gsea/ msigdb).Stemness score analysis was had previously described by Malta et al (1). RNA-sequencing expression profiles and corresponding clinical information for breast cancer were downloaded from the TCGA dataset(https://portal.gdc.com). Use the OCLR algorithm to calculate mRNAsi which constructed by Malta et al. Based on the mRNA expression signature, the gene expression profile contains 11,774 genes. We used the same Spearman correlation (RNA expression data). The minimum value was subtracted, and the result was divided by the maximum maps the dryness index to the range [0,1]. The statistical difference of two groups was compared through the Wilcox test.

**Western blotting and immunoprecipitation**

After extracting the total protein (Beyotime Biosciences, China), 40 μg of protein was electrophoretically transferred to a polyvinylidene fluoride membrane (Millipore). After blocking with 5% skim milk for 2 h, the membrane was incubated with the following primary antibodies at 4℃ for 16 h, Antibodies against TMEM120B(RRID:AB_1845296,Cat#[HPA014066](https://www.proteinatlas.org/ENSG00000188735-TMEM120B/antibody)) were purchased from Sigma-Aldrich (St.Louis, MO, USA) and used at 1:1000 dilutions. anti-OCT4 (RRID: AB_628051

, Cat #5279) was purchased from Santa Cruz Biotechnology Inc(CA, USA)and used at 1:100 dilutions. Anti-integrin-β 1(RRID: AB_448230, ab24693) was purchased from Abcam(Cambridge, Massachusetts, UK) and used at 1:1000 dilutions. Anti-SOX2（AF2018SP）was purchased from R＆D systems(USA) and used at 1:1000 dilutions.Anti-MYH9(Cat#16215-AP-1), anti-α-tubulin (RRID：AB_2210695

,Cat#10094-1-AP,Proteintech, China), anti-ACTN4 (Cat#19096-1-lg) and anti-GAPDH (Cat#600004-1-lg) were purchased from Proteintech (Chicago, IL, USA),aldehyde dehydrogenase 1 (ALDH1, RRID:AB_2044597,Cat#5869)were purchased from R&D systems (Minneapolis, MN, USA).Anti-AKT (RRID：AB_329827,Cat#9272), anti-p-AKT Ser473(RRID：AB_2315049,Cat#4060), anti-TAZ (RRID:AB_2904134,Cat#[72804](https://www.cellsignal.cn/products/primary-antibodies/taz-e9j5a-xp-rabbit-mab/72804?site-search-type=Products&N=4294956287&Ntt=taz&fromPage=plp) and RRID:AB_2800026,Cat#83669),anti-mTOR (RRID:AB_2105622,Cat#2983), anti-p-mTOR (RRID：AB_10691552,Cat#5536), anti-nanog (Cat#4893), anti-YAP (RRID:AB_2650491,Cat#14074 and RRID：AB_2797897,Cat#12395), anti-YAP(RRID:AB_2650491,Cat#[14074](https://www.cellsignal.cn/products/primary-antibodies/yap-d8h1x-xp-rabbit-mab/14074?site-search-type=Products&N=4294956287&Ntt=yap&fromPage=plp)), anti-γ-H2AX(RRID：AB_2118009,Cat#9718), anti-FAK (RRID:AB_2799801,Cat#71433), anti-p-FAK Tyr397 (RRID：AB_10891442,Cat#8556),HA-tag(RRID：AB_1549585,Cat#3724), Lamin B1(RRID:AB_2737428,Cat#13435) ,Flag (RRID：AB_2572291,Cat#14793 and RRID:AB_10950495,Cat#8146) and Myc-tag (RRID：AB_331783,Cat#2276 and RRID：AB_490778,Cat#2278 )were purchased from Cell Signaling Technology (Danvers, MA, USA). Antibodies were used at 1:1000 dilutions. The membrane was washed three times with Tris-buffered saline containing Tween 20 (0.1%) for 10 min. Subsequently, the membrane was incubated with the corresponding secondary antibodies at room temperature for 2 h. Protein bands were visualized using enhanced chemiluminescence.

**MTT and colony formation assay**

MCF-7 and SK-BR-3 or MDA-231 and MDA-453 cells were transfected with TMEM120B, sgTMEM120B and control plasmid for 48 hours. Thereafter, cells were seeded into three 6-cm cell culture dishes (1000 per dish) and incubated for 12 days. Plates were washed with PBS and stained with Giemsa. The number of colonies with >50 cells was counted. For the anchorage-independent colony growth assay, approximately 2000 cells per well were seeded in medium containing 0.4% agarose on top of bottom agar containing 1% low-melting agar in regular medium. After 14 to 21 days, colonies were stained with Giemsa.

TMEM120B-overexpressing MCF-7 and SK-BR-3 or TMEM120B-KO MDA-231 or MDA-453 ~~Cell~~ cells proliferation was evaluated each day for four days after the MTT treatment. The absorbance, which is directly correlated with the number of viable cells in the culture, was measured at 550 nm using a microplate reader (Model 550, Bio-Rad, Hercules, CA, USA). A blank with dimethyl sulfoxide (DMSO) alone was measured and the value was subtracted from all the absorbance for cell culture specimens.

**Wound healing and transwell assay**

Wounds were made with a 200-mL pipette tip when the cultured cells reached a density of <90% confluence. The TMEM120B-overexpressing MCF-7 and SK-BR-3 or TMEM120B-KO MDA-231 or MDA-453 cells were washed to remove cell debris before sub-culturing in 2% serum culture medium. Wound healing within the scrape line was observed at different time points, and representative scrape lines for each cell line were photographed. Duplicate wells for each condition were examined for each experiment, and each experiment was repeated three times. The distance of the wound was optically measured using Image J software.

A cell invasion assay was performed using a 24-well transwell chamber with a pore size of 8 mm (Costar, Corning, NY), and the inserts were coated with 20 mL of 1:3 dilution Matrigel (BD Bioscience). Sixty hours after transfection, TMEM120B-overexpressing MCF-7 and SK-BR-3 or TMEM120B-KO MDA-231 or MDA-453 cells were trypsinized, transferred to the upper Matrigel chamber in 100 mL of serum-free medium containing 3×105 cells, and incubated for 18 hours.

**Transplantation of tumor cells into nude mice**

Four-week-old female BALB/c nude mice were purchased from Slac (Shanghai, China) and mice were housed under pathogen-free conditions in a 12-h dark/light cycle and ad libitum access to food and filtered water. Each mouse was then inoculated subcutaneously with 1×10^6^ of TMEM120B-overexpression SK-BR-3 or control cells in 0.2 mL sterile PBS. Tumor growth was monitored with electronic digital calipers in two dimensions. Six weeks after inoculation, mice were killed by euthanasia and tumors were harvested for future analyses. For chemotherapy treatment, after one-week tumor inoculation, the mice were randomly divided into DMSO+Vector, DMSO+TMEM120B, DMSO+TMEM120B-∆CCD, Docetaxel+Vector, Docetaxel+ TMEM120B, Docetaxel+ TMEM120B-∆CCD, Doxorubicin+Vector, Doxorubicin+ TMEM120B and Doxorubicin+ TMEM120B-∆CCD groups, respectively. Docetaxel and Doxorubicin were administered twice a week at a concentration of 10 mg/kg and 8 mg/kg, respectively. Tumor volume was calculated according to the formula: volume = length × width × width × 0.5. The tumors were fixed in 4% formaldehyde (Sigma), and embedded in paraffin.

To measure the effect on metastatic abilities, 1×10^5^ of TMEM120B-overexpression SK-BR-3 or control cells in 0.1 mL sterile PBS were injected intravenously (tail vein). Eight weeks after inoculation, the mice were euthanized and examined for tumor growth and dissemination. The tumors, hearts, livers, lungs, brain and kidneys were dissected, fixed in 4% formaldehyde (Sigma), and embedded in paraffin. Serial 6 µm-thick sections were cut, stained with H&E, samples were examined with an Olympus CH30 microscope, and images were obtained with a CoolPIX 5400 camera (Nikon, Tokyo, Japan).

REFFERENCES

1. E A Eisenhauer, P Therasse, J Bogaerts, L H Schwartz, D Sargent, R Ford, J Dancey, S, et al. New response evaluation criteria in solid tumours: revised RECIST guideline (version 1.1). Eur J Cancer. 2009;45(2):228-47.

2. Tathiane M Malta, Artem Sokolov, Andrew J Gentles, Tomasz Burzykowski, Laila Poisson, John N Weinstein, et al. Machine Learning Identifies Stemness Features Associated with Oncogenic Dedifferentiation. Cell. 2018;173(2):338-354.
