# Supplementary material for: TMEM120B strengthens breast cancer cell stemness and accelerates chemotherapy resistance via β1-integrin/FAK-TAZ-mTOR signaling axis by binding to MYH9
Source: Breast Cancer Res. 2024 Mar 19;26:48. doi: 10.1186/s13058-024-01802-z (PMC10949598; doi:10.1186/s13058-024-01802-z)
Supplement: Supplementary file 2 — Additional file 2: Primers [file 13058_2024_1802_MOESM2_ESM.docx]

**Table S1. Primers for real-time RT-PCR**

| **Primer sequences (5′→3′)** | |
| --- | --- |
| *TMEM120B* | 5′ GACGCTGTGTAGCAGTTCCATC 3′ |
|  | 5′ GCTCCTTGATGTTCGCTGCCAT 3′ |
| *CYR61* | 5′ CTCGCCTTAGTCGTCACCC 3′ |
|  | 5′ CGCCGAAGTTGCATTCCAG 3′ |
| *CTGF* | 5′ CAGCATGGACGTTCGTCTG 3′ |
|  | 5′ AACCACGGTTTGGTCCTTGG 3′ |
| *TAZ* | 5′ CATGGCTACAAGGACCAGGTTT 3′ |
|  | 5′ GAGGTGCCAGAGCAAATCCA 3′ |
| *MYH9* | 5′ CCT CAA GGA GCG TTA CTA CTC 3′ |
|  | 5′ CTG TAG GCG GTG TCT GTG AT 3′ |
| *GAPDH* | 5′ AAGAAACCCTGGACCACCCAGC3′ |
|  | 5′ TGGTATTCGAGAGAAGGGAGGG3′ |
|  |  |
