# Supplementary material for: TMEM120B strengthens breast cancer cell stemness and accelerates chemotherapy resistance via β1-integrin/FAK-TAZ-mTOR signaling axis by binding to MYH9
Source: Breast Cancer Res. 2024 Mar 19;26:48. doi: 10.1186/s13058-024-01802-z (PMC10949598; doi:10.1186/s13058-024-01802-z)
Supplement: Supplementary file 3 — Additional file 3: Supplementary Figure S1-S8 [file 13058_2024_1802_MOESM3_ESM.docx]

**Supplementary Figures**

**
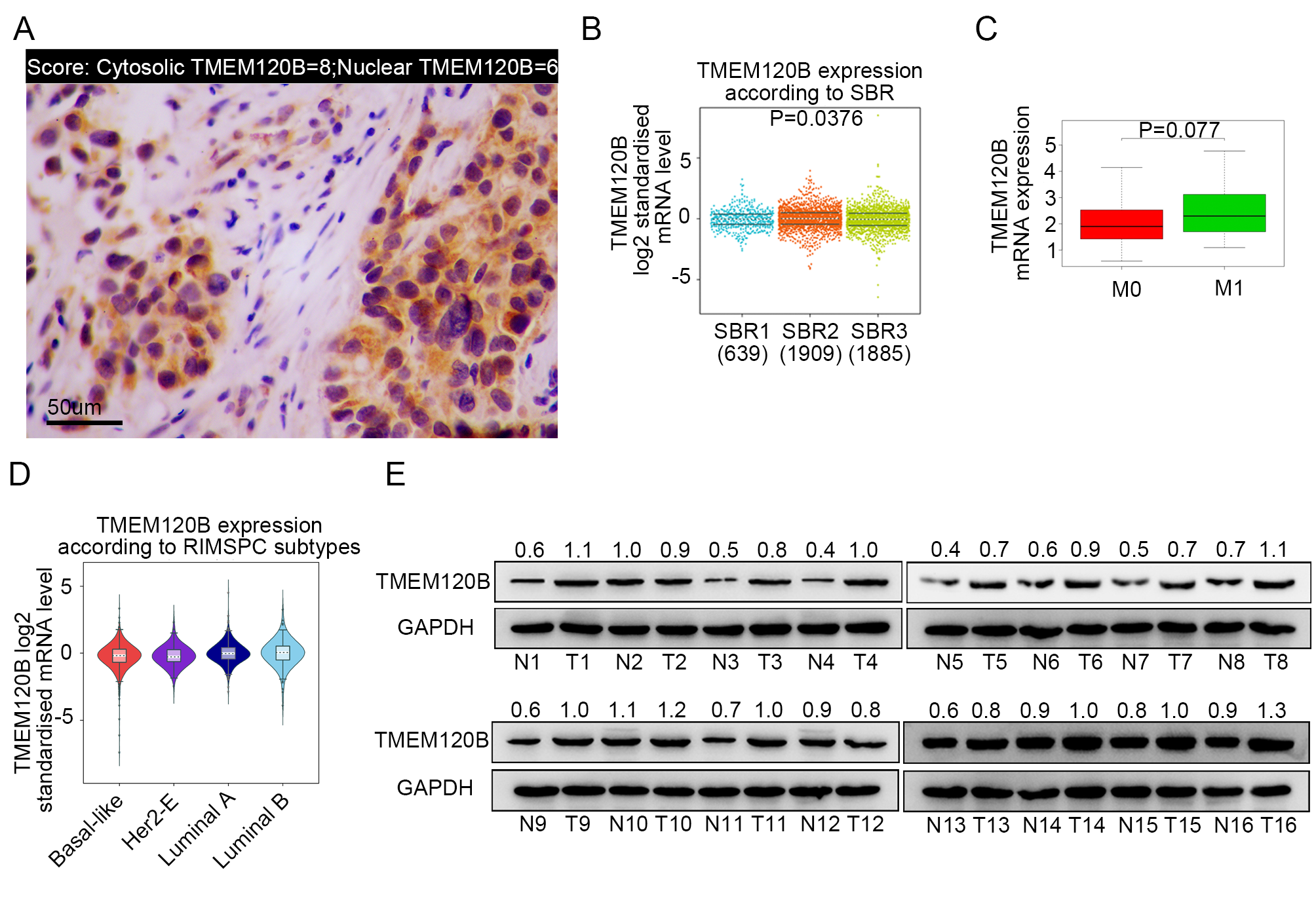
**

**Supplementary Figure 1. TMEM120B was highly expressed in breast cancer specimens**

(A) Representative images of immunohistochemistry staining of nuclear TMEM120B, in human breast cancer specimens(B)TMEM120B mRNA levels were compared between Patients with high or low SBR grades(C) TMEM120B mRNA levels were compared between M0 and M1 in breast cancer patients by using TCGA database(D) TMEM120B mRNA levels were compared among diverse subtypes of breast cancer

(E)TMEM120B protein expression was evaluated by western blotting to 16 pairs of freshly isolated samples from patients with breast cancer

**
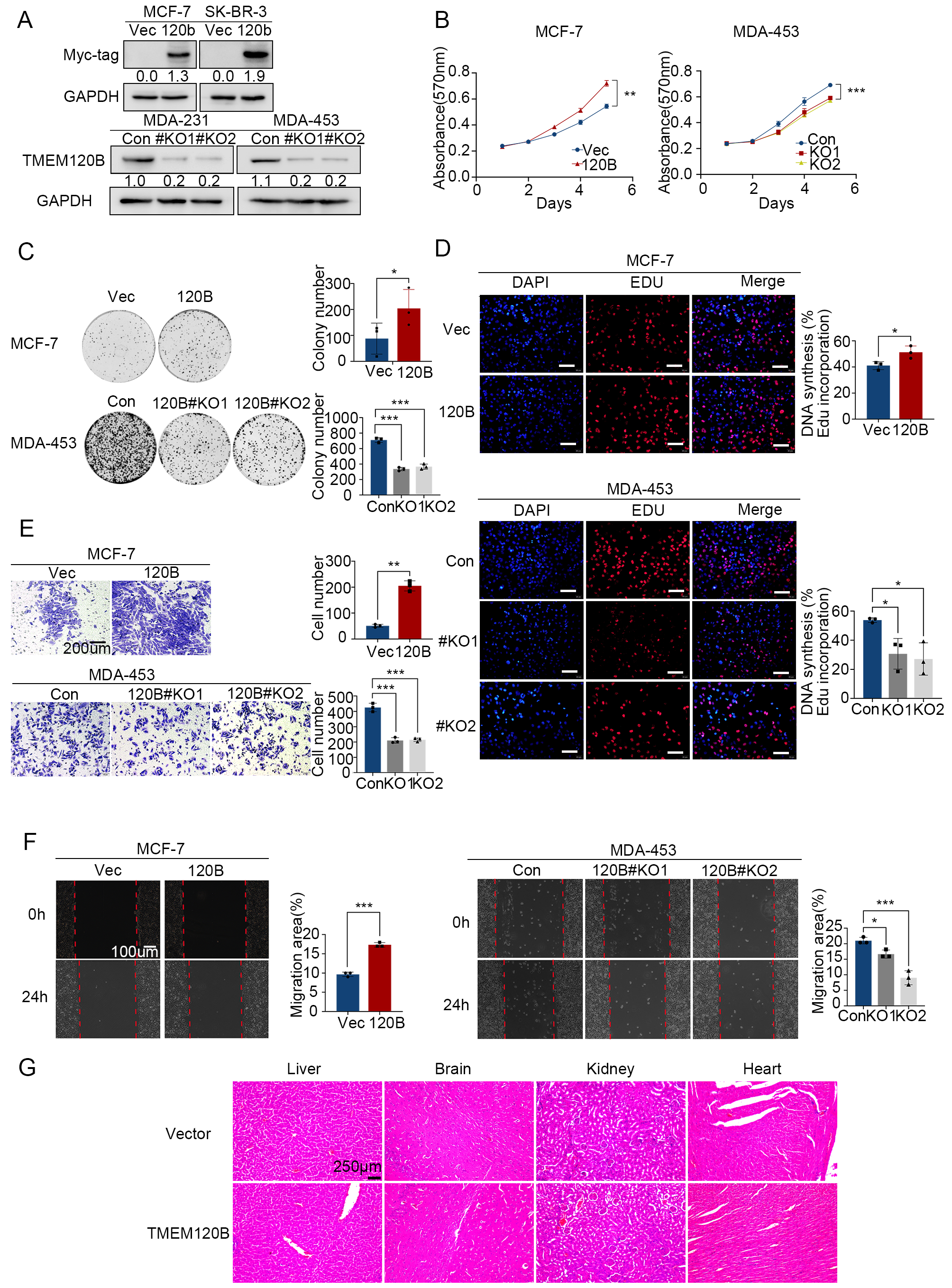
Supplementary Figure S2. Overexpression of TMEM120B promoted breast cancer cells proliferation and invasion both *in vitro* and *in vivo***

1. Overexpression efficiency of TMEM120B were detected in MCF-7 or SK-BR-3 cells and knockout efficiency of TMEM120B was detected in MDA-231 or MDA-453 cells by western blotting. MTT assay (B), colony formation assay (C) and EDU assay (D, scale bar = 100 μm) were performed to examine the effects on the proliferation of breast cancer cells after overexpressing or silencing TMEM120B in MCF-7 or MDA-453 cells. Transwell (E) and wound healing (F) assays were used to check the effects on invasion and migration after transfected with TMEM120B-myc, TMEM120B sgRNA and control in MCF-7 or MDA-453 cells. (G) Representative images of HE staining of metastasis in liver, brain, kidney and heart after overexpressing TMEM120B in SK-BR-3 cells. Quantification data are expressed as Mean ± SD of three independent experiments (t-test, two-sided, *P < 0.05, **P < 0.01, ***P < 0.001).

**
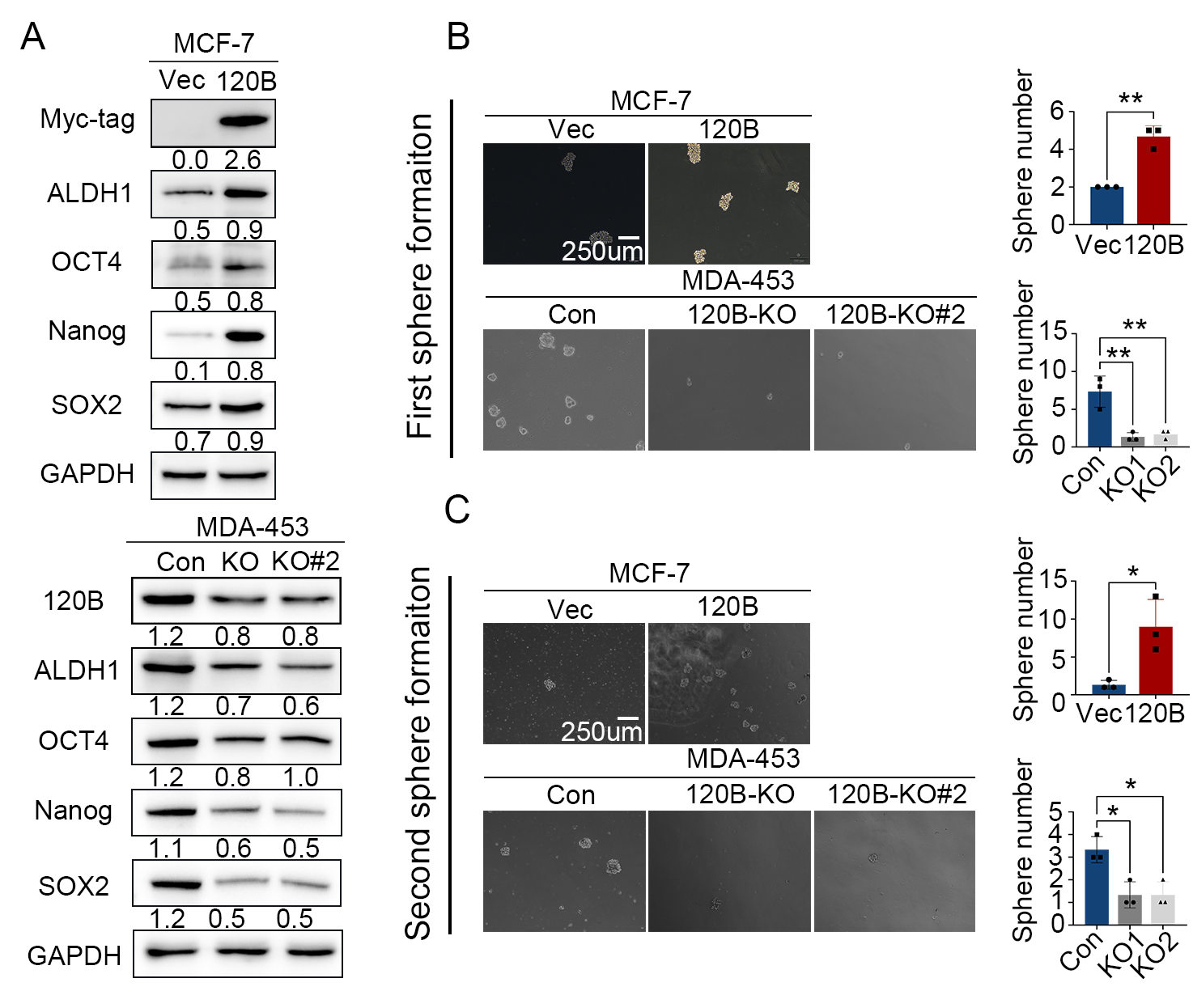
**

**Supplementary Figure S3. Overexpression of TMEM120B enhanced stemness of breast cancer cells**

(A)Immunoblotting of Myc-tag, ALDH1, OCT4, NANOG, SOX2 and GAPDH after overexpressing or deleting TMEM120B in ~~SK-BR-3 cells~~ MCF-7 and MDA-231 cells. Both first (B, scale bar = 250 μm) and second (C) round of sphere formation assays were assessed to examine the effects on stemness of breast cancer cells after overexpressing or knocking out TMEM120B in MCF-7 or MDA-453 cells. Quantification data are expressed as Mean ± SD of three independent experiments (t-test, two-sided, *P < 0.05, **P < 0.01).


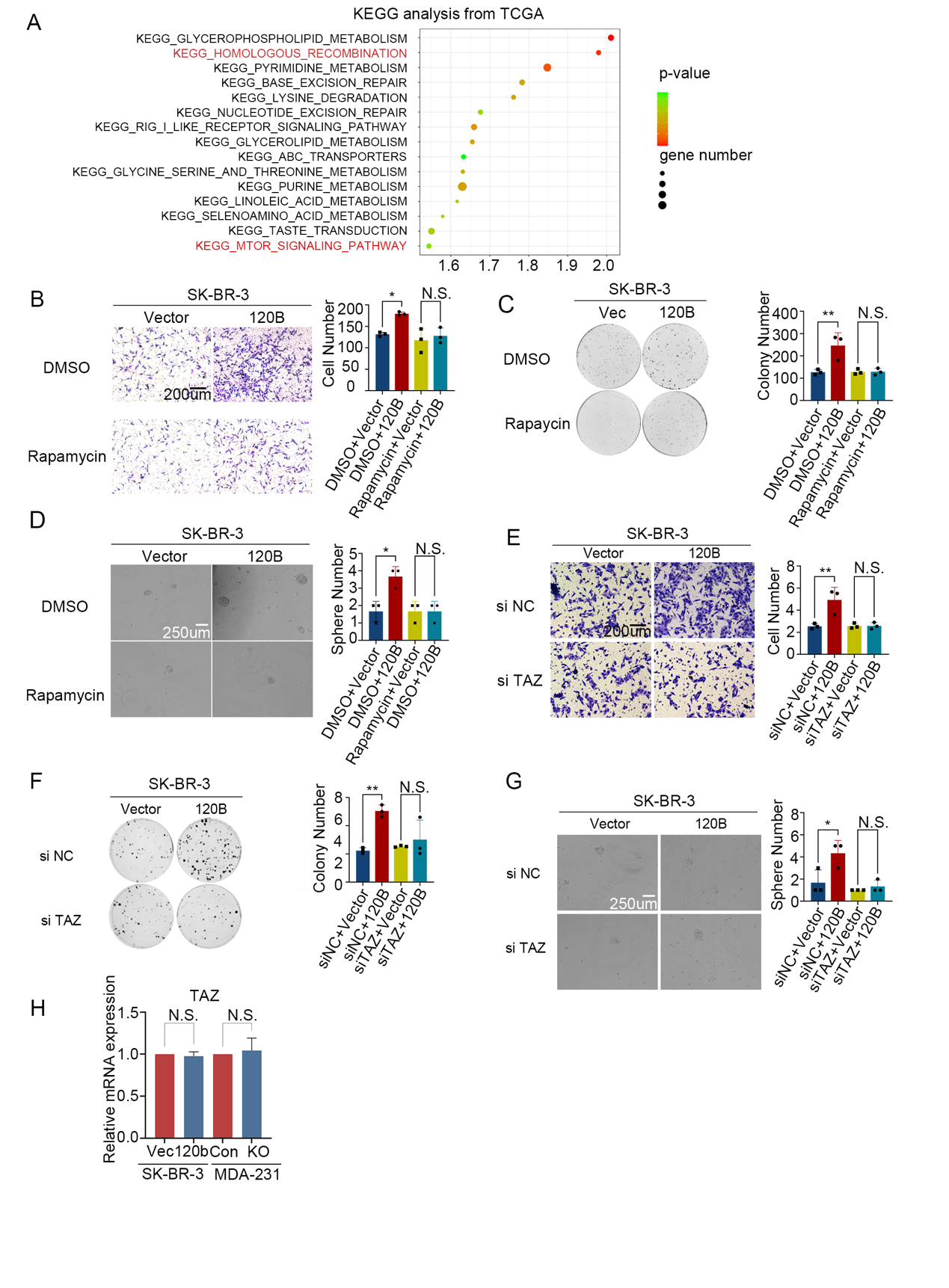


**Supplementary Figure S4. Overexpressing of TMEM120B accelerated breast cancer stemness by activating TAZ-mTOR signaling axis**

(A)Gene set enrichment analysis(GSEA) for TMEM120B silencing. Transwell assay(B), colony formation assay(C) and sphere formation assay(D) were used to detect the effect on the invasion, proliferation and stemness of breast cancer cells after overexpressing TMEM120B with or without mTOR signaling pathway inhibitor Rapamycin in SK-BR-3 cells. Transwell assay(E), colony formation assay(F) and sphere formation assay(G) were used to detect the effect on the invasion, proliferation and stemness of breast cancer cells after overexpressing TMEM120B with or without mTOR signaling pathway inhibitor Rapamycin in SK-BR-3 cells. (H)qPCR assay was used to investigate the alteration of TAZ within ectopic or deleted TMEM120B in SK-BR-3 and MDA-231 cells. Quantification data are expressed as Mean ± SD of three independent experiments (t-test, two-sided, *P < 0.05, **P < 0.01).


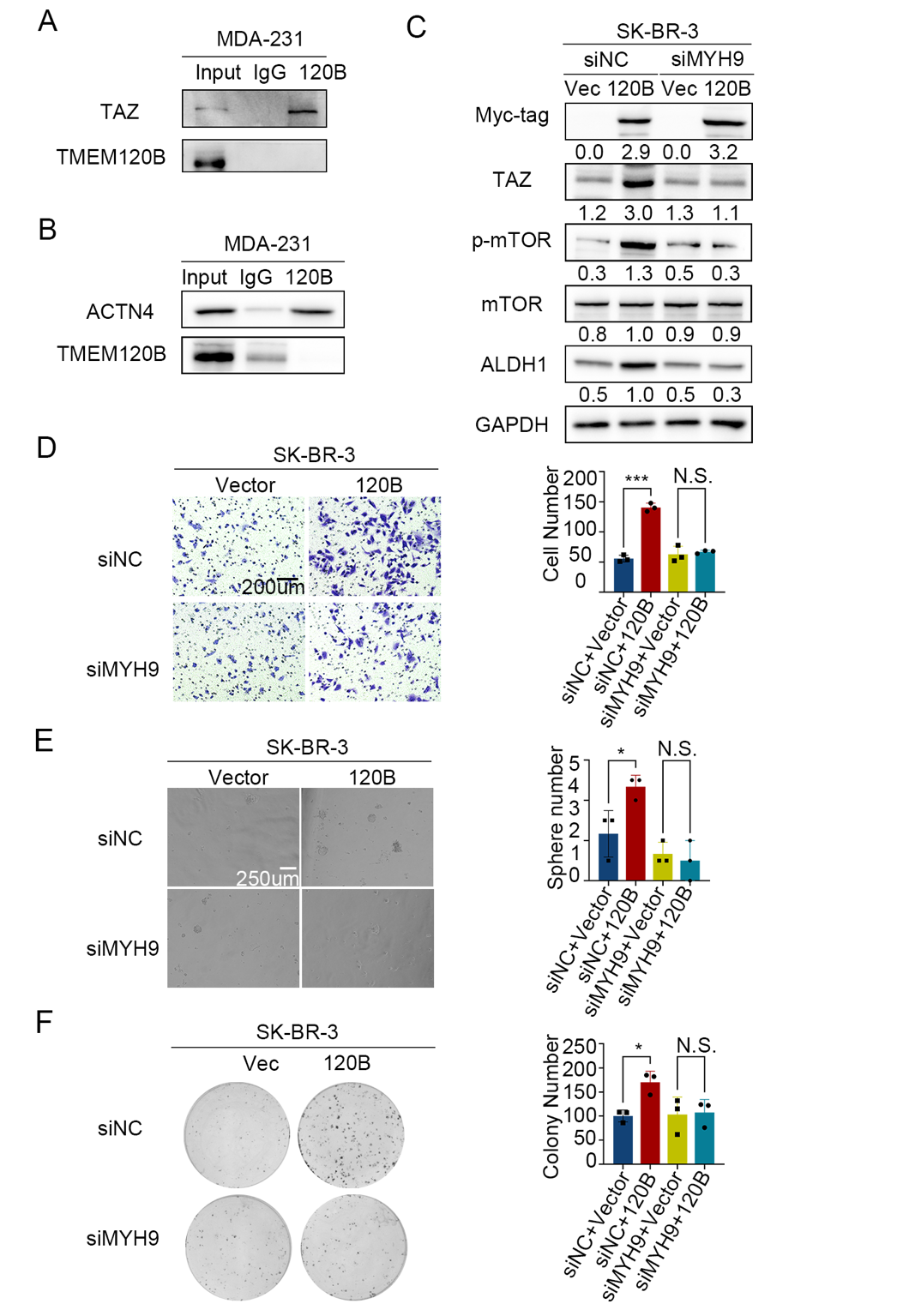


**Supplementary Figure S5. TMEM120B promoted breast cancer cells stemness by binding with MYH9 via their coil-coil domains**

(A-B) Endogenous co-IP assay were assessed to detect the interaction between TAZ, ACTN4 and TMEM120B in MDA-231 cells.(C) Immunoblotting of Myc-tag, mTOR, p-mTOR, TAZ ,ALDH1 and GAPDH after overexpressing TMEM120B with or without siTAZ in SK-BR-3 cells. Transwell assay(D), sphere formation assay(E) and colony formation assay(F) were used to detect the effect on the invasion, stemness and proliferation of breast cancer cells after overexpressing TMEM120B with or without siMYH9 in SK-BR-3 cells. Quantification data are expressed as Mean ± SD of three independent experiments (t-test, two-sided, *P < 0.05, ***P < 0.001).


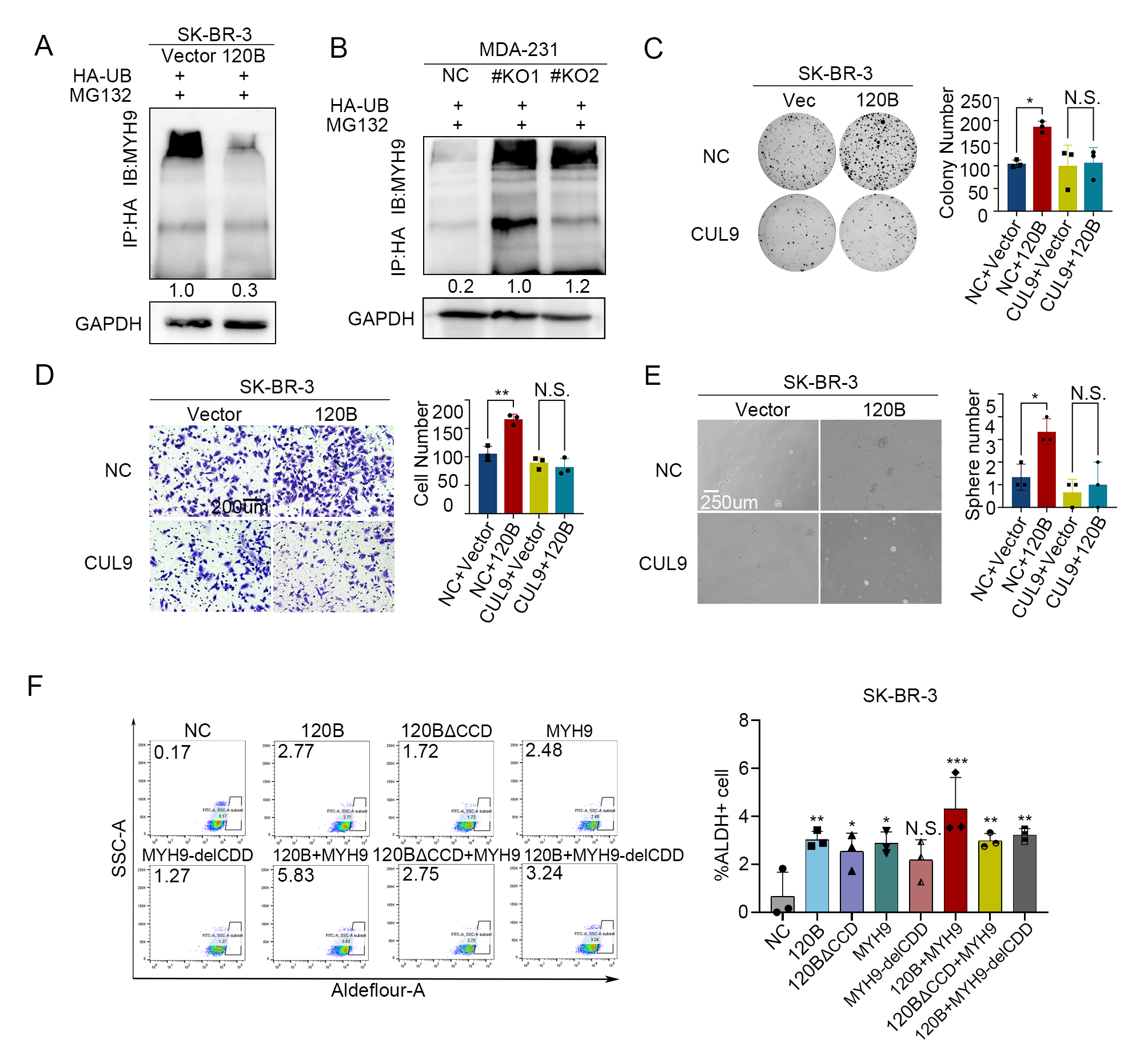


**Supplementary Figure S6. TMEM120B stabilized MYH9 by prevent its ubiquitin-mediated degradation from CUL9**

(A) The ubiquitination level of MYH9 was detected by western blotting after transfected with TMEM120B and control plasmids in SK-BR-3 cells. (B)The ubiquitination level of MYH9 was detected by western blotting after transfected with TMEM120B sgRNA and control in MDA-231 cells. Transwell assay(C), sphere formation assay(D) and colony formation assay(E) were used to detect the effect on the invasion, stemness and proliferation of breast cancer cells after overexpressing TMEM120B with or without siMYH9 in SK-BR-3 cells. (F) Flow cytometry assay detected the ratio of ALDH1+ cells upon overexpressing TMEM120B-myc, TMEM120B-∆CCD-myc, MYH9-flag, MYH9-delCCD-flag alone, or TMEM120B-myc+MYH9-flag, TMEM120B-∆CCD-myc+MYH9-flag, or TMEM120B-myc+MYH9-delCCD-flag in SK-BR-3 cells respectively. Quantification data are expressed as Mean ± SD of three independent experiments (t-test, two-sided, *P < 0.05, **P < 0.01, ***P < 0.001).


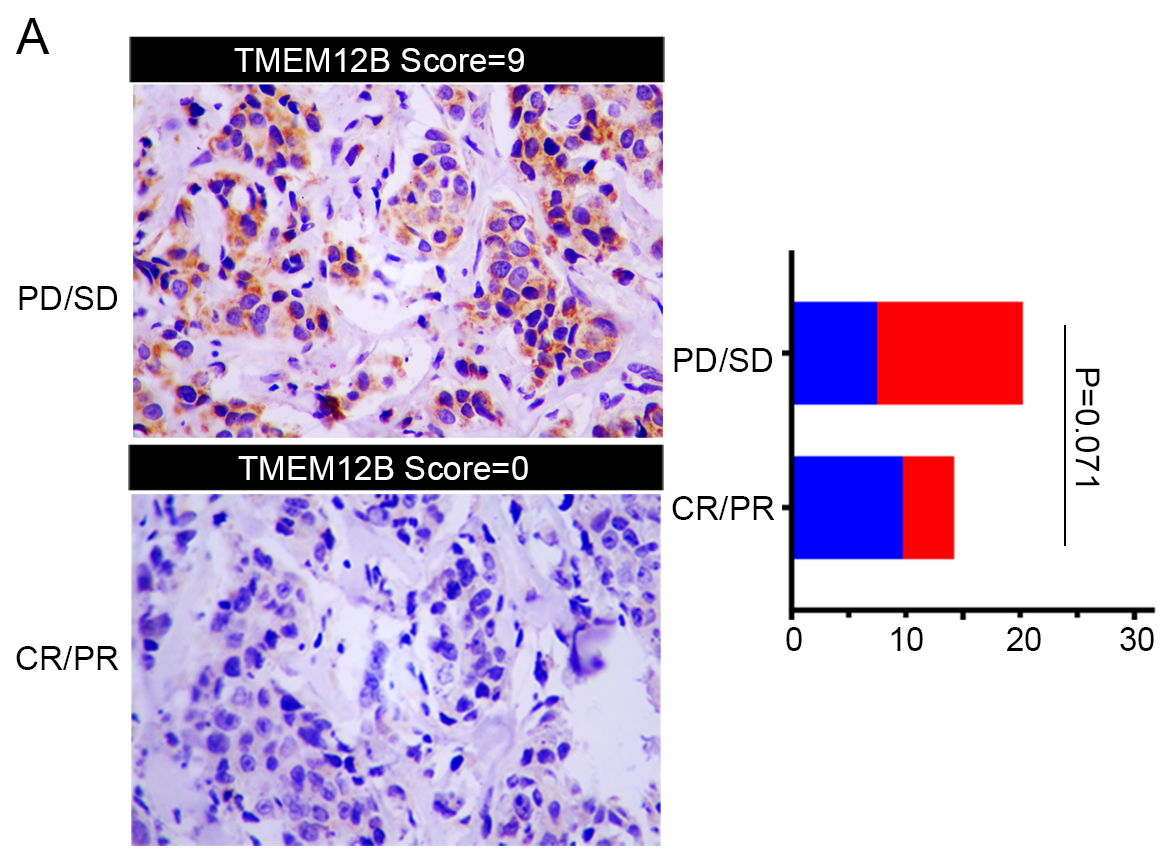


**Supplementary Figure 7. TMEM120B expression significantly positively correlated with chemotherapy resistance in human breast cancer samples**

(A) Representative images of immunohistochemistry staining of nuclear TMEM120B, in human breast cancer specimens with diverse effects upon receiving chemotherapy after surgery.


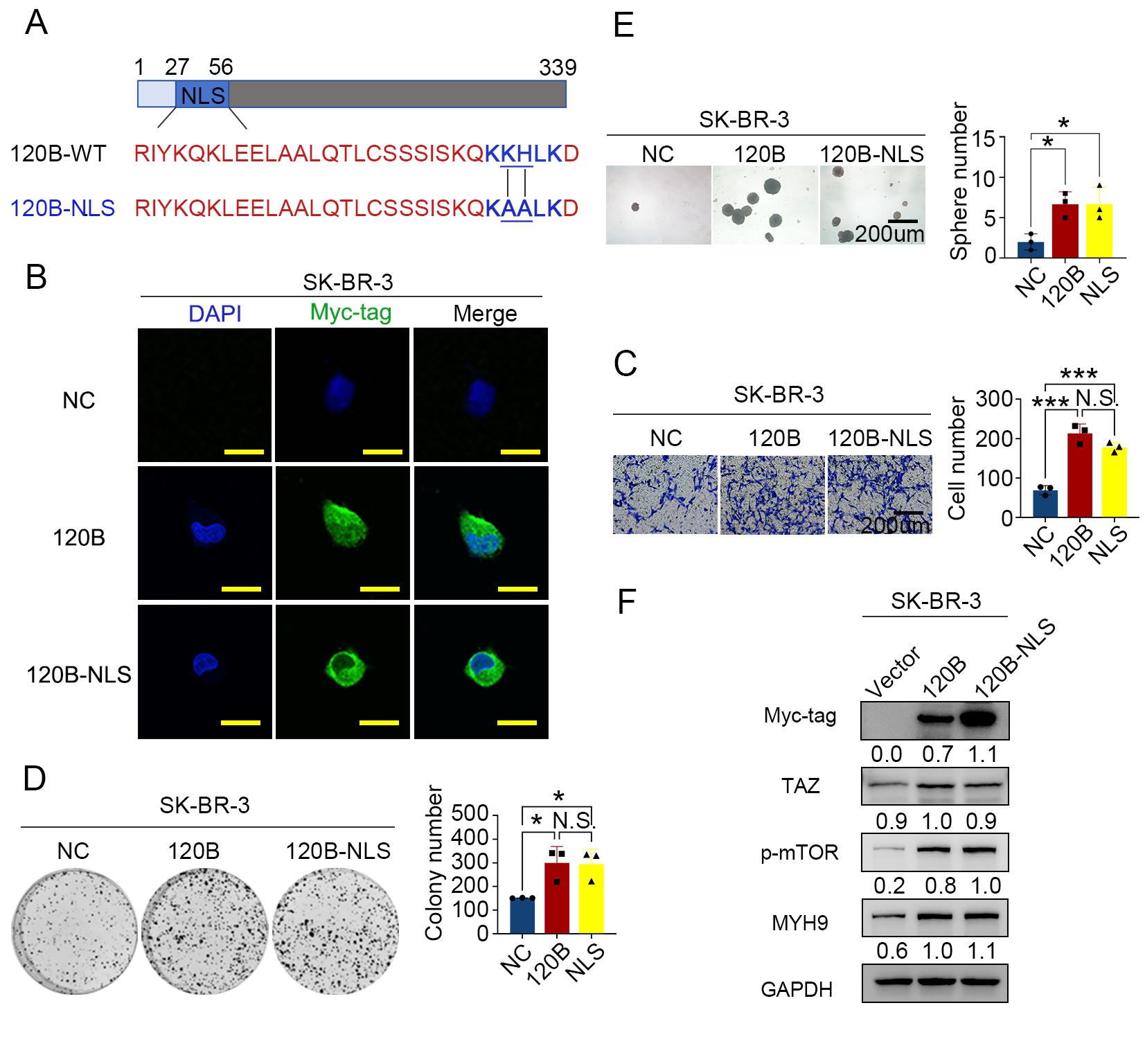


**Supplementary Figure S8. Nuclear TMEM120B is dispensable for elevation of proliferation, invasion and stemness of breast cancer cells**

(A) TMEM120B-NLS mutant plasmids was designed to examine whether nuclear TMEM120B responsible for enhancement of proliferation, invasion and stemness in breast cancer cells (B) Immunofluorescence assay was used to detect the subcellular localization of TMEM120B after overexpressing TMEM120B-WT or TMEM120B-∆CCD plasmids in SK-BR-3 cells (scale bar = 50 μm). Transwell (C), colony formation (E), and sphere formation assays were assessed the effects on proliferation, invasion and stemness after overexpressing TMEM120B-WT, TMEM120B-∆CCD and control plasmids in SK-BR-3 cells (F) Immunoblotting of Myc-tag, p-mTOR, TAZ, MYH9 and GAPDH after overexpressing TMEM120B or TMEM120B-∆CCD in SK-BR-3 cells. Quantification data are expressed as Mean ± SD of three independent experiments (t-test, two-sided, *P < 0.05, ***P < 0.001).
